# Diagnostic utility of whole genome sequencing in adults with B-other acute lymphoblastic leukemia

**DOI:** 10.1182/bloodadvances.2022008992

**Published:** 2023-03-04

**Authors:** Daniel Leongamornlert, Jesús Gutiérrez-Abril, SooWah Lee, Emilio Barretta, Thomas Creasey, Gunes Gundem, Max F. Levine, Juan E. Arango-Ossa, Konstantinos Liosis, Juan S. Medina-Martinez, Krisztina Zuborne Alapi, Amy A. Kirkwood, Laura Clifton-Hadley, Pip Patrick, David Jones, Laura O’Neill, Adam P. Butler, Christine J. Harrison, Peter Campbell, Bela Patel, Anthony V. Moorman, Adele K. Fielding, Elli Papaemmanuil

**Affiliations:** 1 Cancer, Ageing and Somatic Mutation, Wellcome Sanger Institute, Hinxton, United Kingdom; 2 Department of Epidemiology & Biostatistics, Memorial Sloan Kettering Cancer Center, New York, NY; 3 Department of Haematology, University College London (UCL) Cancer Institute, London, United Kingdom; 4 Leukaemia Research Cytogenetics Group, Translational and Clinical Research Institute, Newcastle University, Newcastle upon Tyne, United Kingdom; 5 Cancer Research UK & UCL Cancer Trials Centre, UCL Cancer Institute, UCL, London, United Kingdom; 6 Department of Haemato-Oncology, Barts Cancer Institute, Queen Mary University, London, United Kingdom

## Abstract

Genomic profiling during the diagnosis of B-cell precursor acute lymphoblastic leukemia (BCP-ALL) in adults is used to guide disease classification, risk stratification, and treatment decisions. Patients for whom diagnostic screening fails to identify disease-defining or risk-stratifying lesions are classified as having B-other ALL. We screened a cohort of 652 BCP-ALL cases enrolled in UKALL14 to identify and perform whole genome sequencing (WGS) of paired tumor-normal samples. For 52 patients with B-other, we compared the WGS findings with data from clinical and research cytogenetics. WGS identified a cancer-associated event in 51 of 52 patients, including an established subtype defining genetic alterations that were previously missed with standard-of-care (SoC) genetics in 5 of them. Of the 47 true B-other ALL, we identified a recurrent driver in 87% (41). A complex karyotype via cytogenetics emerges as a heterogeneous group, including distinct genetic alterations associated with either favorable (*DUX4*-r) or poor outcomes (*MEF2D*-r and *IGK::BCL2*). For a subset of 31 cases, we integrated the findings from RNA sequencing (RNA-seq) analysis to include fusion gene detection and classification based on gene expression. Compared with RNA-seq, WGS was sufficient to detect and resolve recurrent genetic subtypes; however, RNA-seq can provide orthogonal validation of findings. In conclusion, we demonstrated that WGS can identify clinically relevant genetic abnormalities missed with SoC testing as well as identify leukemia driver events in virtually all cases of B-other ALL.

## Key Points

• WGS stratifies 88% of B-other ALL in an established genetic subtype that was not possible to detect via cytogenetics.
• Complex karyotype B-ALL emerges as a heterogeneous group of genetic subtypes, including *MEF2D*-r, *DUX4*-r, and *IGK::BCL2*.

## Visual Abstract

**Figure:**
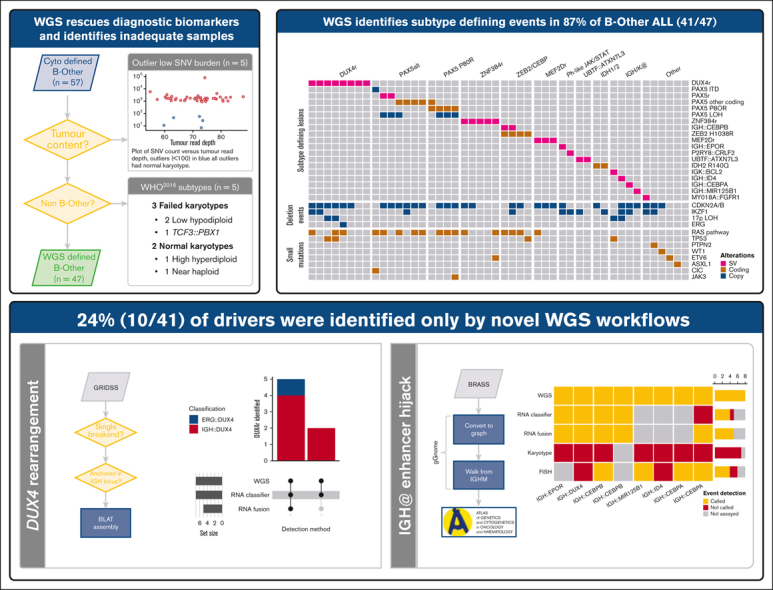

## Introduction

At diagnosis, the genetic classification of adult B-cell precursor acute lymphoblastic leukemia (BCP-ALL) is based on the detection of structural chromosome alterations and altered ploidy states, typically assessed via cytogenetic analysis, fluorescence in situ hybridization (FISH), or reverse transcriptase polymerase chain reaction. These diagnostic strategies inform treatment decisions through the identification of targetable lesions, such as *BCR::ABL1*,^1^ or by assigning patients with high-risk genetic abnormalities, such as *KMT2A* fusion, low hypodiploidy, or complex karyotype to more intensive therapies, such as allogeneic stem cell transplant.^2^^,^^3^

UKALL14 is a UK National Cancer Research Institute Adult ALL group study in which patients were stratified based on the Moorman cytogenetic risk classification.^2^ The outcome for the primary, clinical randomized question has been published recently.^4^ The study also aimed to better understand the relationship between the B-ALL cancer genome, clinical phenotype, and therapeutic response. Genetic profiling of 652 participants with BCP-ALL from the UKALL14 study using cytogenetics, FISH, and multiplex ligation–dependent probe amplification (MLPA) helped confirm the high-risk nature of *KMT2A*-r, low hypodiploid, and complex karyotype and identified JAK-STAT abnormalities as a new high-risk genetic subgroup.^4^ In unison, these profiling approaches assigned 70% of the cases to an established genetic subgroup, leaving 30% of the cases in the undefined and clinically heterogeneous B-other subgroup.

Recent comprehensive profiling approaches using whole transcriptome sequencing have been instrumental in the discovery of disease defining alterations in ALL.^5^, ^6^, ^7^, ^8^, ^9^ In this study, we deploy retrospective whole genome sequencing (WGS) and whole transcriptome sequencing to characterize the genetic landscape of B-other adult ALL. We demonstrate that comprehensive genome profiling allows for the detection of all lesions reported by standard-of-care (SoC) profiling and critically resolve genetic subtypes for the majority of patients with BCP-ALL for whom SoC failed to deliver a definitive diagnosis.

## Methods

### Patients and sample selection

The patients were treated in a randomized trial for adults (age, 25-65 years) with newly diagnosed ALL (UKALL14; ISRCTN66541317; #NCT01085617). Trial participation and correlative research were supported with written informed consent. The study received institutional review board approval. To identify patients with B-other ALL, we performed central review of genetic data collected through SoC testing (cytogenetics, FISH, and reverse transcriptase polymerase chain reaction) as well as research profiling, including FISH (*CRLF2*, *JAK2*, *ABL1*, *ABL2*, and *PDGFRB*) and MLPA (SALSA MLPA Probemix P335, MRC Holland).^10^ A total of 58 cases satisfied the criteria for WGS: (a) absence of genomic drivers (supplemental Figure 1; supplemental Table 1); (b) availability of a diagnostic, pretreatment DNA; and (c) availability of germ line control DNA (minimal residual disease [MRD] <1% or buccal swab). Diagnostic RNA was available for 33 of the 58 cases (supplemental Table 2).

### Sequencing and bioinformatic analysis

WGS was performed (2× 150 bp) to a target read depth of 60× and 30× for the tumor and healthy samples, respectively. RNA sequencing (RNA-seq) was performed using oligo deoxy-thymidine pulldown for a target coverage of 50 million reads (2 × 75 bp).

The WGS and RNA-seq data were aligned to GRCh37d5 using BWA-MEM (supplemental Table 3) and STAR version 2.5.0c (supplemental Table 4). WGS variant calling proceeded with 57 of 58 sequence complete pairs to determine somatic single nucleotide variants (SNVs), insertions/deletions, copy number aberrations, and structural variants (SVs).^11^, ^12^, ^13^, ^14^, ^15^ Variants were annotated with OncoKb, to determine their putative role in cancer pathogenesis.^16^

Data from 33 RNA-seq cases were analyzed to classify ALL subtypes using the consensus of any 2 of the ALLSorts,^17^ ALLSpice,^18^ and ALLCatchR (https://github.com/ThomasBeder/ALLCatchR) classifiers. Sample clustering with the City of Hope B-ALL reference gene expression data set from the MD-ALL R package (https://github.com/gu-lab20/MD-ALL) was used to finalize subtype classification. RNA fusions were detected using FusionCatcher^19^ and CICERO.^20^ RNA mutations in PAX5 (R38H/C, P80R, and R140L) and ZEB2 (H1038R) were manually checked using Integrative Genomics Viewer.^21^ Data integration, analysis execution, and visualization were conducted using the Isabl platform.^22^

To detect enhancer hijacking events associated with the *IGH* locus, we used gGnome (https://github.com/mskilab/gGnome) to construct a graphical representation of the SVs detected in the tumor sample. For each graph, the gGnome “proximity” function was used to “walk” from the Eμ *IGH* superenhancer locus to genes in GENCODE version 29.^23^^,^^24^ Candidate IGH walks <100 kbp were annotated using the Atlas of Genetics and Cytogenetics in Oncology and Haematology and Mitelman Database of Chromosome Aberrations and Gene Fusions in Cancer.^25^^,^^26^

For DUX4r detection, we used the GRIDSS^27^ SV caller, which allows for the identification of single breakends (SVs unambiguously anchored at only 1 locus). For each single breakend identified, BLAT was used to map the unplaced sequence to hg19.^28^ Single breakend sequences mapped to chrUn_gl000228 (an unplaced hg19 contig with DZ4Z repeats containing *DUX4*) were manually reviewed using Integrative Genomics Viewer.^21^ For further details please refer to supplemental Methods.

### Mutational signatures

For single base substitutions (SBSs), mutation signature analysis was performed using the R/Bioconductor MutationalPatterns (version 3.5.6) package^29^ using a 7-signature SBS mutational catalog as reference (supplemental Figure 3).^30^ RAG-mediated deletions were annotated via motif analysis using Multiple Em for Motif Elicitation (MEME) https://meme-suite.org/meme/tools/meme.^31^^,^^32^

### Statistical analysis

R version 3.6.3 (29 February 2020) was used; Mann-Whitney *U* test was used for between groups comparison of continuous variables using the wilcox.test function.^33^ A robust linear model was used to calculate the per year SNV burden using the rlm function from the MASS R package,^34^ and 95% confidence intervals using the confint.default function.^33^

## Results

**Figure 1.**
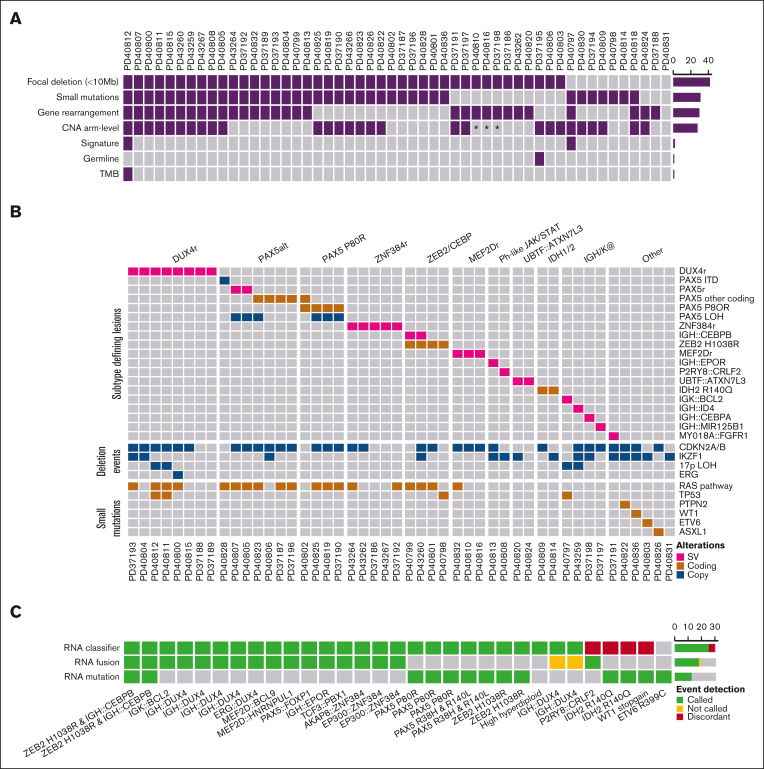
**Comprehensive genome profiling using WGS.** (A) Heatmap describing the presence of genomic markers detected via WGS in 52 cases, with samples in columns and markers in rows: (a) focal deletions (<10 Mb) in tumor suppressor genes, (b) small mutations (SNV/insertion and deletion) in cancer genes, (c) recurrent gene rearrangements seen in ALL, (d) arm-level CNA, (e) outlier SBS mutational signature profile, (f) germ line variant, and (g) outlier tumor mutation burden (TMB). (B) Oncoplot of true B-other cases (n = 47) showing subtype defining lesions and recurrent deletion events and genes recurrently targeted by small mutations (SNVs/INDELs) in OncoKB or putative drivers identified in the “other” category. (C) Comparison of WGS-defined subtypes with RNA expression subtype classification and RNA fusion calling in 31 cases with both DNA and RNA data available. ∗ denotes samples with suboptimal copy number profiles; ITD, internal tandem duplication.

Comprehensive profiling via WGS characterized arm-level and focal copy number alterations (CNAs), SVs, and acquired somatic and germ line mutations, mapping at least 1 aberrant somatic lesion in 52 of 57 B-other ALL samples in our cohort (Figure 1A). The remaining 5 samples were excluded from subsequent analysis because of a low mutation burden (<100; supplemental Figures 4 and 5), suggesting a low tumor burden. Notably, all 5 low purity samples were erroneously considered, via cytogenetics, to have normal karyotypes (supplemental Table 6). This resulted in a set of 52 cases, of which 31 had RNA-seq data as well (supplemental Table 7).

Arm-level CNAs were the most common class of alteration (n = 139; median 1), followed by focal deletions (<10 Mb) in tumor suppressor genes (n = 106; median 2), and acquired somatic mutations in cancer genes (n = 61; median 1). Most cases (81%; 42/52) had focal deletions, 73% (38/52) had acquired somatic mutations targeting established cancer genes, 58% (30/52) had recurrent gene rearrangements, and 57% (28/52) harbored arm-level CNAs (Figure 1A).

### Genomic classification of B-other cases by WGS

WGS analysis of the 52 B-other cases identified 5 (10%) cases, with an established World Health Organization (WHO) 2016 genetic subtype that was missed by SoC testing (supplemental Table 8): 3 were high-risk (2 low hypodiploid and 1 near haploid) cases, and 2 were standard-risk (high hyperdiploid and *TCF3::PBX1*) cases. Cytogenetic analysis of these 5 cases either failed (n = 2) or produced a normal karyotype (n = 3). The median inferred tumor purity for these cases was 46% (range, 33%-96%), compared with 89% (range, 13%-99%) for the remaining patients (n = 47). Of note was the near triploidy case (PD37195), with a germ line *TP53* (p.N239D, 17:7577566 T>C; variant allele frequency [VAF] in healthy tissue = 0.29; tumor VAF = 0.87 with chr17 copy neutral loss of heterozygosity [LOH]) mutation (supplemental Table 8).

Among the remaining 47 B-other cases, WGS analysis helped identify abnormalities, enabling 41 (87%) cases to be classified into the following subtypes: *DUX4* rearrangements (*DUX4*-r, n = 8), PAX5alt (n = 7), PAX5 P80R (n = 4), *ZNF384* rearrangements (*ZNF384*-r, n = 5), *ZEB2*/*CEBP* (n = 4), *MEF2D* rearrangements (*MEF2D*-r, n = 3), *UBTF::ATXN7L3* fusions (n = 2), *IDH1*/*2* mutations (n = 2), and Ph-like JAK-STAT abnormalities (n = 2), along with single cases of *IGK::BCL2*, *IGH::CEBPA*, *IGH::ID4*, and *IGH::MIR125B1* (Figure 1B; supplemental Figure 6; supplemental Table 8). With the exception of *IGH* rearrangements and ZEB2 H1038R, these genetic lesions define nonoverlapping subgroups. Deletions targeting *CDKN2A*/*B*, *PAX5*, and *IKZF1* were prevalent across the genetic subtypes. The same was true for mutations targeting RAS/MAPK signaling genes (*NRAS*, *KRAS*, *FLT3*, *NF1*, *PTPN11*, *GNB1*, and *EGFR*; supplemental Table 8; Figure 1B).

The remaining 6 (13%) B-other cases could not be classified into a recognized B-other subtype (supplemental Table 9). Case PD37191 harbored a *MYO18A::FGFR1* fusion [t(8;17)(p11;q23)], which most likely maps to the WHO 2016 myeloid/lymphoid neoplasms with FGFR1 rearrangement classification.^35^ Two cases (PD40822 and PD40836) involved clonal frameshifts in genes associated with T-cell ALL (*PTPN2* p.A108fs∗5 and *WT1* p.V371fs∗14, respectively). PD40803 involved a somatic *ETV6* p.R399C previously reported as a germ line risk^36^ with a concurrent complex SV within *KDM6A* (supplemental Table 10), and 1 case harbored a clonal mutation typically associated with myeloid disease *ASXL1* p.R417∗ (PD40826). Lastly, WGS failed to identify a putative driver in 1 case (PD40831), with an estimated tumor purity of 28% (supplemental Table 9).

### Comparison of WGS and RNA-seq subtype allocation

To evaluate the diagnostic utility of WGS compared with that of RNA-seq, we compared the diagnostic findings in 31 cases using both WGS and RNA-seq data (supplemental Table 7). WGS analysis identified a disease defining genetic alteration in all 31 cases (Figure 1C; supplemental Table 8). Of the 31 cases with RNA-seq data, 5 were classified as low RNA sample quality: 3 cases (PD37187, PD43260, and PD43262) with evidence of cross contamination (supplemental Table 7), and 2 cases (PD37188 and PD40803) identified as low purity that impaired gene expression–based classification (supplemental Figure 7A; supplemental Table 11). Estimation of the percent blast count via RNA and WGS correlated well, with the expected exception of the high hyperdiploid case (supplemental Figure 7B).

In 19 of the 31 cases, the underlying lesion contained genomic rearrangement. Evaluation of RNA-seq classification based on gene expression and fusion detection (supplemental Tables 11 and 12) correctly classified 19 cases (16 with both, 1 fusion only, and 2 classifiers only; supplemental Table 13). Fusion detection missed 2 *IGH::DUX4* rearrangements. In a single case with *P2RY8::CRLF2* (PD40808), RNA classification was split between Ph-like (ALLCatchR and ALLSorts) and iAMP21 (City of Hope reference data set). In this case, WGS analysis only identified a single chr21 gain rather than a high-level amplification,^37^ which was consistent with the diagnostic karyotype (50,XY,+21,+22 inc/46,XY).

In 3 cases (PD37187, PD43260, and PD43262), additional RNA fusions were not concordant with the consensus RNA classifier subtype (supplemental Table 12), which were the same samples identified using Somalier with cross contamination (supplemental Table 7).

Of the 13 cases, defined by coding gene mutations (4 × *ZEB2* H1038R, 3 × *PAX5* P80R, 2 × PAX5alt, 2 × *IDH2* R140Q, 1 × *ETV6* R399C, and 1 × *WT1* frameshift), RNA classification was assigned to 12 of 13 cases leaving the *ETV6* R399C case (PD40803) unclassified in both WGS and RNA-seq. Of the 12 classified cases, 9 were concordant between the WGS and RNA-seq–derived subtypes (supplemental Table 13). Of the 3 discordant cases, 2 had *IDH2* R140Q mutations (PD40809 and PD40814), and 1 had a frameshift WT1 mutation (PD40836). All 3 cases were classified as PAX5alt by 3 of the 4 RNA classifiers; in comparison, 3 cases with bona fide PAX5 alterations were classified as PAX5alt by 4 of the 4 classifiers (Figure 1C; supplemental Tables 11 and 13). Lastly, the B-other case with *ETV6* R399C had an additional *KDM6A::CGA* rearrangement identified via RNA-seq fusion detection, which was not predicted via WGS fusion calling.

### Resolution of cytogenetic findings

**Figure 2.**
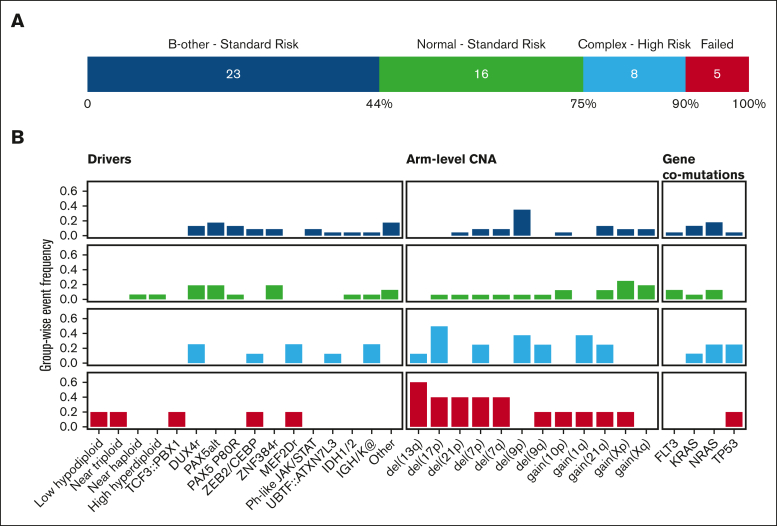
**Resolution of cytogenetic findings.** Resolution of cytogenetic findings in the 52 cases. (A) Bar plot of the SoC cytogenetic classification. (B) Somatic event profile per cytogenetic group describing the occurrence of driver, arm-level CNAs, and coding comutations (y-axis denotes group-wise frequency).

Cytogenetics analysis of the cohort (n = 52) revealed a normal (n = 19), nonspecific abnormal (n = 23), complex (n = 8), or failed karyotype (n = 2). These cytogenetic categories represent highly heterogeneous genetic subgroups (Figure 2A). WGS analysis identified a driver event in 100% (8/8) of the cases with a complex karyotype, classifying them into 1 of 6 different genomic subtypes: *DUX4*-r, *MEF2D*-r, *ZEB2*/*CEBP*, *UBTF::ATXN7L3*, *IGK::BCL2*, and *IGH::ID4* (Figure 2B). There was no correlation between complex karyotype and genomic subtype; however, 2 of 3 *MEF2D*-r cases with cytogenetics had a complex karyotype, and 3 of 8 cases had a TP53 mutation with 17p loss (2 × *DUX4*-r and 1 × *IGK::BCL2*; supplemental Table 8; supplemental Figure 8). The same was true for cases with normal, nonspecific abnormal, or failed karyotypes, in which we identified diverse genetic lesions, including *DUX4*-r, *ZNF384*-r, and *ZEB2*/*CEBP* among others. Notably, 56% (9/16) of cases with normal karyotypes had either a cytogenetically cryptic genomic rearrangement (7/16) or an altered ploidy class (2/16).

### Demographic, clinical, and genetic features of B-other genomic subtypes

Most *DUX4*-r cases harbored the canonical *IGH::DUX4* rearrangement (7/8), but only 1 case had *ERG::DUX4* (PD40815; supplemental Table 8). Only a single concurrent intragenic *ERG* deletion was identified (PD40800a; 21:39773785-39875948), which has been reported in up to two-thirds of cases with pediatric *DUX4*-r ALL^38^ (supplemental Figure 9). Interestingly, 6 of 8 (75%) patients with *DUX4*-r were female compared with 17 of 44 in the study cohort(39%). Unlike prior observations in both pediatric and adult ALL, suggesting that *DUX4*-r was associated with favorable outcome,^9^^,^^39^ only 1 of 8 were MRD-positive at the end of induction, and 5 of 8 relapsed (supplemental Figure 10).

We identified 11 cases with *PAX5* abnormalities mapping to PAX5alt (n = 7) and PAX5 P80R (n = 4) subtypes. Notably, 10 of the 11 cases showed evidence of biallelic targeting by either 2 mutations (n = 4) or a single mutation/fusion and concomitant LOH (n = 6; supplemental Table 8; supplemental Figure 11). Both PAX5alt fusions had corresponding karyotypic events: *ETV6::PAX5* was detected via cytogenetics as dic(9;12)(p13;p13), and *FOXP1::PAX5* presented as add(9)(p13). The other 5 PAX5alt contained 3 cases with R38 and R140 biallelic mutations, 1 case harboring an intragenic *PAX5* amplification (supplemental Figure 12A), and 1 with *PAX5* deletion and a protein truncating mutation (p.M335fs∗68;supplemental Figure 12B). We also detected 4 *PAX5* P80R: 3 with arm-level LOH and 1 with a secondary *PAX5* R38H mutation (supplemental Figure 13). Although monoallelic *PAX5* deletions were observed across genomic subgroups, both PAX5alt and *PAX5* P80R were demarcated by a dependency on biallelic targeting (supplemental Table 8; supplemental Figure 6).

Five cases had a *ZNF384* fusion, including *EP300::ZNF384* (n = 4) and *AKAP8::ZNF384* (n = 1). We recently reported that patients with *ZNF384* are typically younger and have good outcomes.^10^ As expected, the same is true for patients in this subset. Four of 5 (80%) were aged ≤40 years compared with 18 of 47 (40%) for the rest of the cohort; only 1 of 5 were MRD-positive at the end of induction, and 2 of 5 relapsed (supplemental Figure 10).^10^

WGS analysis identified 4 cases belonging to the *ZEB2*/*CEBP* group: 2 cases with *ZEB2* H1038R and concurrent *IGH::CEBPB* rearrangements and 2 cases with ZEB2 H1038R alone. A fifth *ZEB2* H1038R mutation was detected in an *IGH::DUX4* case (PD40800; VAF = ∼0.20); however, RNA-seq classified this sample as *DUX4*-r, and the remaining 4 ZEB2 H1038Rs were all classified as *ZEB2*/*CEBP* (supplemental Tables 11 and 13).

**Figure 3.**
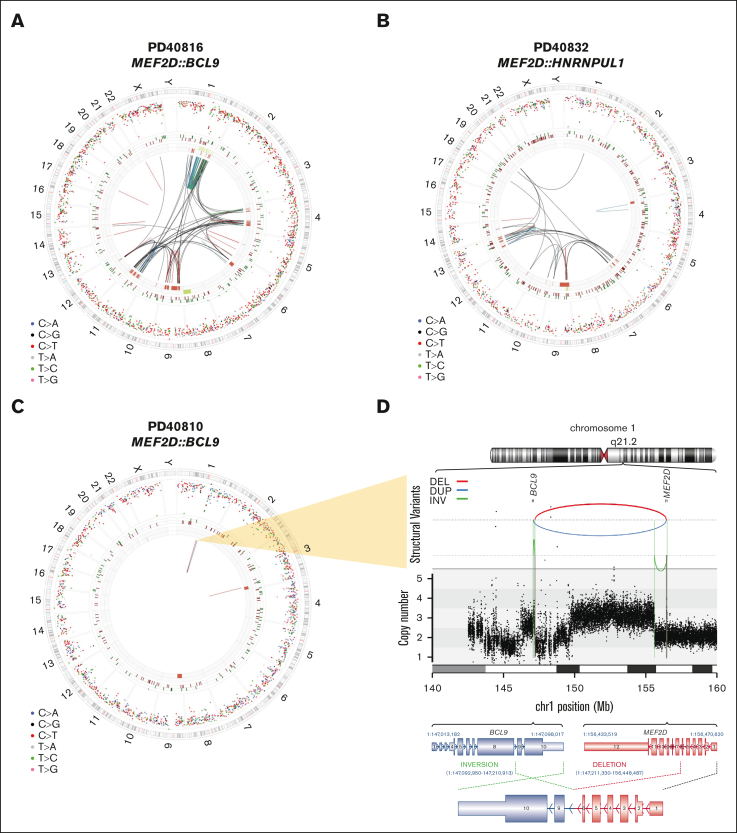
**Complexity of MEF2D genomes in B-ALL.** Genomic landscape of MEF2D-rearranged B-ALL. (A-B) Circos plots showing high SV burden in the 2 MEFDr cases with chromothripsis. The circos plot shows 4 concentric panels, and the outermost ring shows the chromosome ideogram. The second ring shows the intermutation distance for all SNVs color-coded by the pyrimidine partner of the mutated base (C>A, blue; C>G, black; C>T, red; T>A, gray; T<C, green; and T>G, pink). The third ring shows small insertions (dark green) and deletions (dark red). The fourth ring shows copy number change gains (green) and losses (red). Intra- and interchromosomal SVs are shown by arcs describing translocations (black), inversions (blue), deletions (red), and duplications (green). Circo plot (C) and locus plot of 1q21 (D) showing the “quiet” global SV burden and complex causative local SV event in an *MEF2D::BCL9* case. In the locus plot in panel D are shown SV events as arcs between 2 breakpoint loci, with the color denoting the type of SV (top); the absolute copy number (middle); and the 2 causative SVs (inversion and deletion) overlaying the transcript structure of *BCL9* and *MEF2D* (bottom). DEL, deletion; DUP, duplication; INV, inversion.

Of the 3 *MEF2D*-r cases identified, 2 cases had complex karyotypes (PD40816, *MEF2D::BCL9*; and PD40832, *MEF2D::HNRNPUL1*) and exhibited copy number oscillation involving multiple chromosomes (chr1, 4, 9, and 11 and chr6, 9, and 13, respectively), indicative of chromothripsis (Figure 3A-B). The remaining case of *MEF2D::BCL9* (PD40810a) failed cytogenetics and had a relatively stable genome (Figure 3C). However, *MEF2D::BCL9* rearrangement was a consequence of a highly complex localized SV, involving an interstitial jump into an intergenic region (Figure 3D). Notably, all 3 *MEF2D*-r cases exhibited copy gain of *MEF2D*, which confounded the initial detection by FISH (supplemental Figure 14). All 3 *MEF2D*-r cases were classified as high risk in UKALL14: 2 based on complex karyotype and 1 based on age. In keeping with this high-risk status, all 3 *MEF2D*-r cases relapsed (supplemental Figure 10).

We previously screened patients in this cohort for ABL-class fusions and JAK-STAT abnormalities using FISH and MLPA (supplemental Figure 1). However, WGS revealed 2 additional cases of Ph-like JAK-STAT abnormalities. The first had *IGH::EPOR*, which is not detectable via FISH or MLPA; whereas, the second had *P2RY8::CRLF2* fusion, which was validated via FISH and MLPA.^10^

Four cases harbored newly described subtypes; two cases (PD40820 and PD40824) had a *UBTF::ATXN7L3* fusion resulting from a submicroscopic interstitial deletion at 17q21.31. Both cases also harbored the 13q12.2 deletion, which hijacks the *PAN3* enhancer to drive overexpression of *CDX2* (supplemental Figure 15).^40^^,^^41^ Two more cases (PD40809 and PD40814) harbored clonal *IDH2* p.R140Q mutations (supplemental Figure 16), a subtype recently described by Yasuda et al.^41^

Lastly, 13 cases involved the hijacking of an immunoglobulin gene enhancer (*IGH* x12 and *IGK* x1). In addition to the aforementioned *DUX4*-r (n = 7) and *ZEB2*/*CEBP* (n = 2) cases, WGS analysis revealed 4 additional cases of *IGK::BCL2*, *IGH::CEBPA*, *IGH::ID4*, and *IGH::MIR125B1*. The patient with *IGK::BCL2* (PD40797) was classified to be at high-risk (age, >40 years; complex karyotype) and, although achieving remission after induction, responded poorly to therapy dying shortly after diagnosis (<200 days). The detection of this abnormality at the initial diagnosis might have prompted the re-evaluation of the diagnosis.

### Detection of DUX4 rearrangements

**Figure 4.**
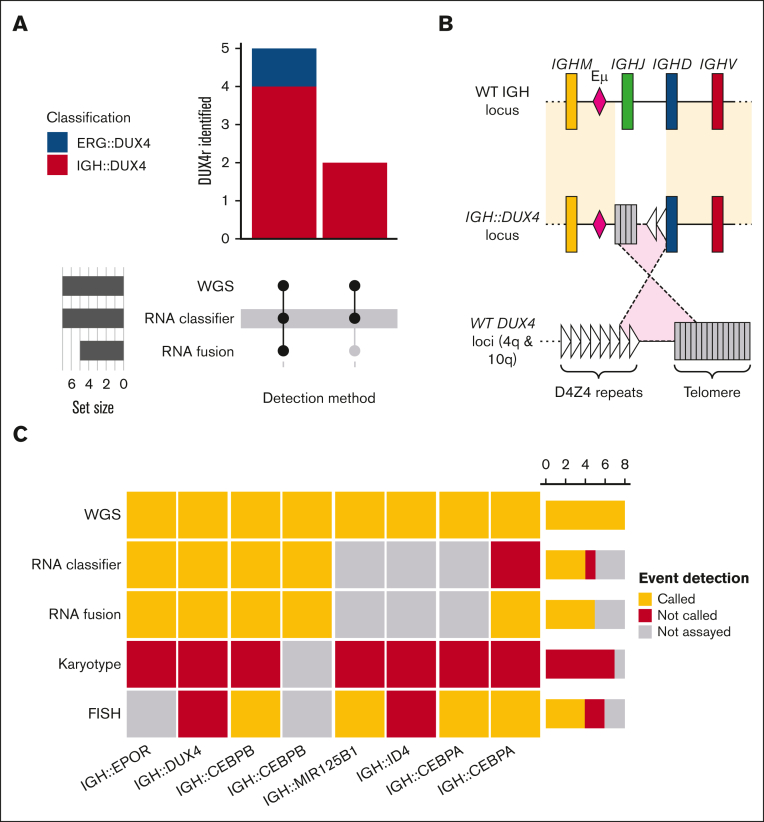
**Identification of *DUX4* and *IGH@* rearranged by WGS.** (A) UpSet plot detailing *DUX4*-rearranged event identification in 7 cases with both WGS and RNA data. (B) Graphical representation of a complex *IGH::DUX4* locus configuration detected via WGS in only 2 cases (PD37188 and PD40804), which confounds the RNA fusion calling. The upper and lower loci represent the wild-type (WT) *IGH* locus and *DUX4* loci (4q and 10q). The middle represents the *IGH::DUX4* loci, where an inverted insertion of the *DUX4* loci, including the proximal telomere into the IGH locus, confounds the RNA fusion calling. (C) Heatmap of *IGH*@ events identified using WGS and correlative evidence from RNA fusion calling, RNA expression classification, and cytogenetics (karyotype and FISH).

Using our custom workflow, 8 rearrangements targeting the *DUX4* locus were identified (supplemental Table 14). RNA-seq data were available for 7 of the 8 cases, allowing for the comparison of WGS with RNA fusion and RNA classification analyses (Figure 4A). RNA classification identified all 7 cases, but RNA fusion gene analysis failed to detect 2 *IGH::DUX4* cases (PD37188a and PD40804a). WGS showed evidence that in both cases the 5′ IGH locus translocation mapped to telomeric repeats, whereas the 3′ IGH translocation mapped to the *DUX4* loci. Therefore, we can infer that, in these 2 cases, the *DUX4* locus along with proximal telomeric repeats was inserted into the IGH locus in an inverted orientation, which confounds detection by RNA fusion calling (Figure 4B). The *ERG::DUX4* (PD40815a) case also showed expression of an alternative exon 6 (supplemental Figure 17), as previously described.^38^ WGS analysis and *DUX4* gene expression analysis, but not RNA fusion analysis, showed high specificity for the detection of *DUX4*-r. The implementation of WGS enables the detection of all *DUX4* rearrangements and provides resolution on the structure of both the rearrangement and partner genes.

### Detection of *IGH* enhancer rearrangements

Using the graph-based *IGH* enhancer hijack calling workflow, we reclassified 8 SV calls as candidate *IGH* events (supplemental Table 15). Partner genes included recurrent events in *DUX4*, *EPOR*, *CEBPA*, and *CEBPB* and the less common *MIR125B1* and *ID4*. Seven *IGH* rearrangements were directly validated via FISH or RNA-seq fusion analysis, and 1 case (PD43259) had indirect evidence from FISH and karyotype, showing a 14q32 deletion correlating to the loss incurred due to an unbalanced translocation to form *IGH::ID4* (supplemental Table 15; Figure 4C). Finally, in terms of the sensitivity of this approach, this workflow identified an *IGH* rearrangement that was validated via both RNA-seq and FISH in a case that failed to meet the purity criteria (20%) for WGS analysis (PD40837, *IGH::CEBPA*; supplemental Table 15).

### Detection of focal deletions

**Figure 5.**
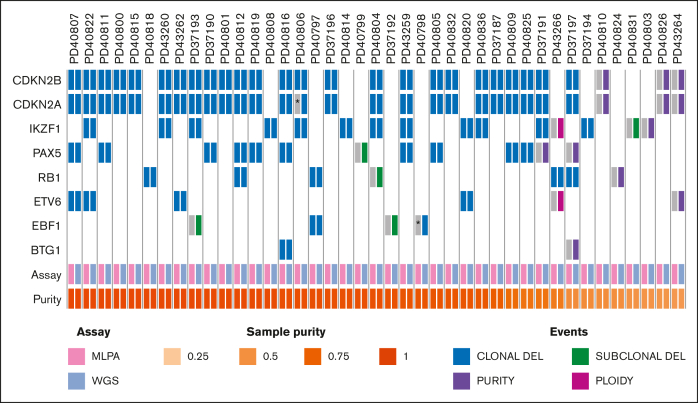
**MLPA and WGS copy number comparison.** Tile plot comparing deletions across MLPA (P335) target genes, called via MLPA and WGS (ASCAT and BRASS). For each case, the events recovered by the MLPA and WGS are shown as paired columns. Events missed by MLPA are marked by a gray tile. ∗ denotes the 2 events missed owing to the MLPA probe placement.

Focal deletions in *EBF1*, *IKZF1*, *CDKN2A*, *CDKN2B*, *PAX5*, *ETV6*, *BTG1*, and *RB1* are strongly associated with ALL pathogenesis. For 48 cases, high-quality MLPA and WGS CNA data were available for these 8 gene targets.^10^ MLPA detected 82 deletions in 32 cases (median deletion count, 3; range, 1-4). WGS analysis was 100% concordant by detecting all 82 deletions characterized via MLPA (Figure 5). Importantly, WGS analyses detected an additional 21 deletions missed by MLPA in 15 cases; most of these deletions were attributed to sample purity/ploidy (n = 14) or a subclonal event (n = 5), with the final 2 events not identified via MLPA because of probe placement (Figure 5; supplemental Table 16).

### Patterns of genomic instability in BCP-ALL

Assessment of the genome-wide patterns of mutations present in each leukemia genome allows for the characterization of putative biological processes or environmental exposures that result in genomic instability and contribute to leukemic transformation.^31^^,^^42^

**Figure 6.**
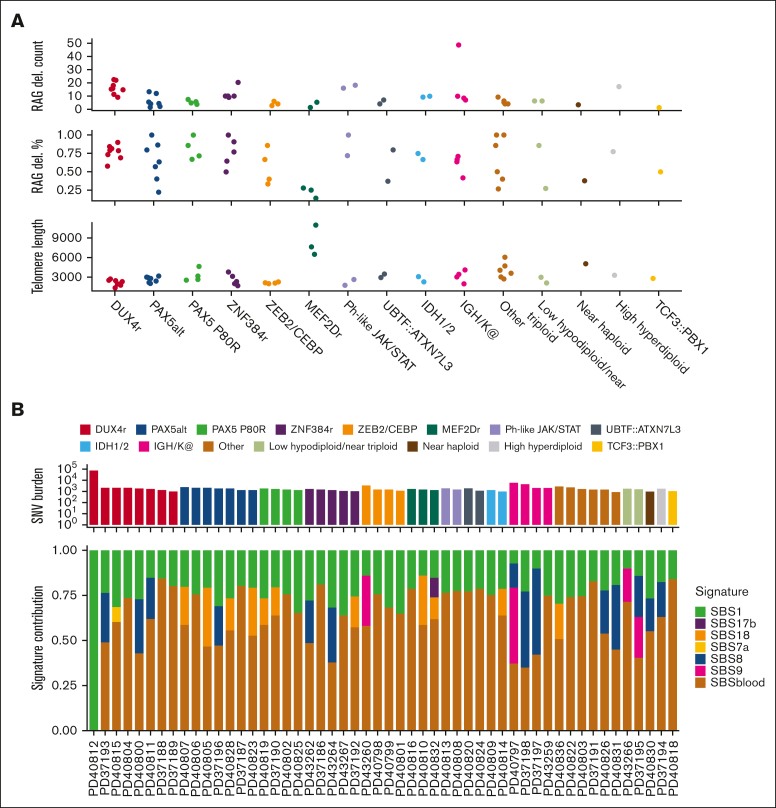
**Mutational patterns.** (A) Dot plot showing the RAG burden (total count and percent of all deletions) and telomere length for each subtype. (B) Plot showing the total SNV burden (top) and the normalized SBS signature exposure (bottom). (C) 96 context mutational profiles of SBS1, SBS9, SBS18, and SBS blood.

SV analysis identified B-cell–specific processes, such as RAG-mediated deletions,^31^ and observed complex SV events, such as chromothripsis.^43^ RAG-mediated deletion were identified in all cases, with a median burden of 7 events (range, 1-49). A high burden of RAG-mediated deletions was observed in subtypes *DUX4*-r and *ZNF384*-r (Figure 6A). The highest RAG-mediated deletion burden was identified in a single *IGH::MIR125B1* case (PD37197), with 49 of 69 deletions attributable to RAG activity (supplemental Figure 18). The subtype with the lowest contribution to RAG-mediated deletions was *MEF2D*-r, which correlates with the previous observation that *RAG1* is downregulated in *MEF2D*-r.^44^ We also observed outlier high telomere length in *MEF2D*-r (Figure 6A).

Analysis of SBS patterns for the established mutation signatures revealed that the majority of SNVs were mapped to the SBS blood signature (Figure 6B-C). SBS blood is a clock-like signature operative in hematopoietic stem cells enriched in C>T mutations.^30^^,^^45^ SBS blood was detected in 51 of 52 samples in our cohort and demonstrated a linear relationship with age, explaining ∼16 mutations per year of life (supplemental Figure 19). The only exception was an *IGH::DUX4* case with homozygous loss of *MSH6* that was defined by hypermutation (PD40812 with 77 183 SNVs) enriched in SBS1, a phenotype previously observed in hypermutator cases.^46^^,^^47^ Although the sample mutational profile was atypical for SBS1 (Cosine similarity = 0.941), a much better fit was the thio–mismatch repair deficient signature (Cosine similarity = 0.996; supplemental Figure 20A-B). A signature observed in cases of relapsed pediatric hypermutator ALL has been recently attributed to those of mismatch repair–deficient ALL treated with thiopurine.^48^^,^^49^ The patient had a history of Crohn disease, in which thiopurine administration is a common therapy. SBS9 is a signature dominated by T>G and T>C mutations attributed to replication errors by polymerase η during somatic hypermutation in lymphoid cells.^50^ In our cohort, SBS9 was observed in 4 cases: 2 low hypodiploid cases, 1 *IGH::CEBPB* and 1 *IGK::BCL2* case with the highest mutation burden (42%), which we had expected, given the association of this lesion with mature B-cell neoplasms. Lastly, SBS18, a signature dominated by C>A mutations and attributed to reactive oxygen species, was enriched in the *MEF2D*-r, *PAX5*, and *IDH1*/*2* subtypes.

## Discussion

The diagnostic work up for adult ALL does not incorporate WGS in most of the centers worldwide. Here, we assessed the utility of WGS profiling in 52 adult ALL cases, that in the absence of informative biomarkers by SoC were classified as B-other.

WGS assigned 88% (46/52) of the cases called B-other to an established genetic subtype of ALL, with ∼20% (10/46) of subtypes being assigned solely via the novel WGS workflows developed in this study. This included 5 cases with WHO 2016 subtypes; 3 of the 5 cases harbored high-risk genetic events that would have changed their UKALL14 risk group and postinduction treatment in the absence of other risk factors. Among the remaining B-other cases, 87% (41/47) were assigned to 1 of the newly described genetic subtypes of ALL.^9^^,^^39^^,^^41^ Our recent studies have proposed that several of these subtypes are linked to a good (eg, *ZNF384*-r) or poor (eg, JAK-STAT) outcome.^10^^,^^39^

Our findings demonstrate that unless cytogenetics reveals an established genetic rearrangement or ploidy subtype, a designation of failed, normal, or complex karyotype frequently misses subtype defining events that can readily be picked up by WGS, as was also demonstrated in our related studies on childhood ALL.^51^^,^^52^ For example, the presence of a complex karyotype, defined as ≥5 chromosomal abnormalities, has been associated with a poor prognosis.^2^^,^^10^ WGS identified a driver event in all 8 cases, revealing a heterogeneous spectrum of drivers linked both to favorable (*DUX4-*r) and poor (*MEF2D*-r and *UBTF::ATXN7L3*) outcomes. This suggests that a complex karyotype is not a robust classification.

The WGS also identified events that could alter clinical management. One *IGH::DUX4* case involved a hypermutation caused by homozygous loss of *MSH6*, a candidate for checkpoint inhibition. A second case involved homozygous loss of *CD58* and concurrent LOH of *HLA-B,* which would likely confer immune escape^53^^,^^54^ and reduce the efficacy of chimeric antigen receptor T-cell therapy.^55^^,^^56^ Lastly, a near triploidy case harbored a germ line *TP53* mutation, which has implications for carrier screening.

Integration of RNA-seq for fusion gene detection and gene expression classification enables concomitant validation of WGS based findings using an orthogonal assay. This is of particular importance for transcriptomically defined phenocopy subtypes, such as Ph-like or PAX5alt, for which WGS alone would only be able to evidence previously defined recurrent DNA lesions. RNA-seq can also directly identify fusions caused by complex SVs that are missed in standard WGS fusion calling.

Because this was a retrospective study, we used remission samples to source germ line DNA. To avoid tumor contamination, we selected samples with MRD-negative results or MRD <1%. Therefore, the observed genomic subtype frequencies may not reflect the true distribution in adult B-ALL. In this study, we focused on cases that did not harbor informative clinical biomarkers and were classified as B-other. We did not formally assess the performance of WGS and RNA-seq against SoC in patients for whom the ALL subtype was previously determined.

For clinical implementation, detailed laboratory analytical and clinical validity studies are warranted to delineate standardization metrics for diagnostic assay deployment and the optimal source of normal DNA to determine assay performance against SoC molecular diagnostic assays across the spectrum of B-ALL subtypes.

Conflict-of-interest disclosure: G.G. is a consultant at Isabl Inc. M.F.L. is an employee and equity holder at Isabl Inc. J.S.M.-M. is the founder and equity holder at Isabl Inc. A.A.K. has received honoraria from Kite. L.C.-H. on behalf of the Cancer Research UK and University College London Cancer Trials Centre has received research funding from 10.13039/100004325AstraZeneca, GSK, 10.13039/100004319Pfizer, MSD, Bristol Myers Squibb, Amgen, and Millennium Takeda. P.C. is a cofounder, stock holder, and consultant for FL86 Inc. B.P. is on the advisory board for Pfizer. A.V.M. has received honoraria from Amgen. A.K.F. has served as a consultant for Amgen, Pfizer, and Novartis. E.P. is a founder, equity holder, and holds a fiduciary role in Isabl Inc and is an equity holder in TenSixteen Bio. The remaining authors declare no competing financial interests.

The current affiliation for S.L. is St George’s University Hospitals NHS Foundation Trust, London, United Kingdom.

The current affiliation of K.Z.-A. is The Royal London Hospital, Barts Health NHS Trust, London, United Kingdom.

## Supplementary Material

Supplemental Tables

Supplemental Methods, Tables, Figures, and References
