# Supplementary material for: Diagnostic utility of whole genome sequencing in adults with B-other acute lymphoblastic leukemia
Source: Blood Adv. 2023 Mar 4;7(15):3862–73. doi: 10.1182/bloodadvances.2022008992 (PMC10405200; doi:10.1182/bloodadvances.2022008992)
Supplement: Supplemental Methods, Tables, Figures, and References [file BLOODA_ADV-2022-008992-mmc2.pdf]

Diagnostic utility of whole genome sequencing in adults with B-other acute lymphoblastic leukemia

Supplementary material

Supplementary Methods

Molecular profiling

Molecular Diagnostic G-banded karyotype and Fluorescence in situ hybridization (FISH) were performed locally by each regional center. Subsequently these were centrally reviewed at the Leukaemia Research Cytogenetics group (Newcastle University)[1]. Additionally, multiplex ligation-dependent probe amplification (MLPA) was performed using the P335 ALL-IKZF1 kit (MRD Holland, The Netherlands), at the Leukaemia Research Cytogenetics group, Newcastle University, as previously described[2]. An expanded FISH screen (*CRLF2*, *JAK2*, *ABL1*, *ABL2* & *PDGFRB*) was also performed on B-other cases to identify Ph-like fusion cases at the Leukaemia Research Cytogenetics group, Newcastle University[1].

At time of selection MLPA screening was undertaken using the coffalyser analysis program at WTSI[3].

Sample selection

For WGS, cases were selected which had a diagnostic DNA and a MRD <1% follow-up sample or buccal swab, to be used as control. Additionally diagnostic timepoint RNA was also selected for RNA-seq if available.

There were 394 B-ALL cases which met the sample requirements, 221 were classed as B-Other using SoC cytogenetics. These were further screened with the additional research FISH panel and MLPA (P335) to enrich for non Ph-like cases, this yielded 89 cases eligible for sequencing (S. Figure 1).

WGS library preparation, sequencing and alignment

1. Library preparation and Sequencing

Prior to library preparation, DNA samples underwent SNP genotyping by custom fluidigm panel to facilitate sample matching between tumor and normal sample pairs. DNA was sheared to a target size of 450bp and paired-end libraries prepared for sequencing Illumina X Ten platform. PCR-free workflow was used if sufficient input DNA was available (>= 1ug) otherwise, the standard PCR workflow was used with 500ng input. There were 10 library failures, all normal buccal swab samples and replacements were found for 9/10 affected samples; 4 samples from cases with MRD markers (3 MRD level was <1% and 1 "Indeterminate" sample with QR 1e-3) and 5 samples from cases without

identified MRD markers (Supplemental Table 2). The library type (standard/no PCR) of each final sample and average coverage is detailed in Supplemental Table 3.

2. Alignment

FASTQ files were aligned to the 1000genomes Phase2 Reference Genome Sequence (hs37d5), using BWA-MEM (v0.7.15 & v0.7.17).

ftp://ftp-trace.ncbi.nih.gov/1000genomes/ftp/technical/reference/phase2_reference_assembly_sequence/hs37d5.fa.gz

WGS analysis

1. Post-alignment QC

Sequencing coverage estimates were measured by the sum of mapped read pairs length in base pairs (bp) divided by the aligned reference in bases (GRCh37d5 3,137,282,682 bp) (Supplemental Table 3).

Assessment of read depth showed no significant difference between PCR and PCR-free library preparation workflows between tumor (Supplemental Figure 2a) and normal samples (Supplemental Figure 2b).

After alignment we used Somalier[4] to confirm sample identity between WGS tumor normal pairs. All pairwise comparisons were below 0.5 relatedness suggesting no appreciable contamination (Supplemental Table 7).

2. Variant calling pipelines

WGS tumor and normal paired analysis was performed using the Wellcome Sanger Institute pipeline, which has been extensively validated (https://github.com/cancerit).

The following algorithms were used for each variant type, with variant filters applied as standard in this study.

SNV

- CaVEMan (v1.11.2/1.13.14)[5]
    - Filter
        - PASS variants
        - ASRD>=0.93 (read-length adjusted quality of reads)
        - CLPM==0 (mutations occurring on reads which have been clipped)

INDEL

- Pindel (v2.2.2/2.2.4/2.2.5/3.2.0)[6]
    - Filter
        - PASS variants
        - S1 > = 30 (simple score of support reads (“# +” + 1)* (“# -” + 1))
        - Normal sample VAF < 0.1

CNV

- ASCAT (v4.0.1/4.1.2/4.2.1)[7]
- Battenberg (v3.0.1/3.3.0/3.2.2/3.3.1)[8]

SV

- BRASS (5.4.1/6.1.2/6.0.5/6.2.0/6.3.0)[9]
    - Filter
    - (copynumber_flag == 1 | !is.na(assembly_score)) & readpair.count > 6

3. Assessment of tumor-in-normal (TiN) contamination

TiN was assessed using the TINC R package[10]

i. Input was CaVEMan PASS calls along with any SNVs flagged by the inbuilt TiN filters MN and MNP.
ii. Calls were then filtered with ASRD>=0.93 and CLPM==0.
iii. Autosomal calls were kept and reformatted for TINC.
iv. TINC was run only with SNVs.

4. Purity and ploidy determination

To determine tumor purity and ploidy prior to running CaVEMan and BRASS, we used a two-step heuristic;

i. If the initial estimates of ASCAT and Battenberg agreed these were finalized.
ii. Otherwise, MLPA and the median INDEL VAF from PINDEL were used to guide refitting of purity and ploidy in both algorithms.

Finalized purity ploidy solutions were reviewed and categorized as (WGS Ploidy solution in Supplemental Table 7);

- OK: solution was good fit
- Sub-optimal: finalized solution was deemed sub-optimal

Three cases had "Sub-optimal" solutions, so we excluded them when reporting specific copy number features.

- PD37198 - had discordant initial estimates and a very “noisy” WGS copy number profile confounding an optimal solution. The solution was finalized with the initial Battenberg fit which matched the CaVEMan VAF peak nearest to 0.5 (~0.07) giving a sample purity of ~13%.

- PD40810 - had discordant initial estimates and a “noisy” WGS copy number profile and a low number of copy number events. The solution was finalized with the initial ASCAT fit which matched the CaVEMan VAF peak nearest to 0.5 (~0.16) giving a sample purity of ~30%.

- PD40816 - had concordant initial values but the initial purity estimate (~61-64%) was discordant with the CaVEMan VAF peak nearest to 0.5. The solution was finalized to the purity inferred by the CaVEMan VAF peak (~0.47) ~92%.

5. Sample QC

We also sought to ascertain which cases may have low diagnostic potential due to either.

- Cross contamination identified by Somalier (relatedness > 0.5)
- Tumor sample purity < 20% (ASCAT)
- Normal sample tumor in normal >= 1% (TINC)
- Sub-optimal purity ploidy solution

This was coded as “High” or “Low” in the “WGS sample quality” column in Supplemental Table 7 & Supplemental Table 8.

6. Cancer gene annotation

To augment the standard variant annotation, we used OncoKB to prioritize cancer associated genes, using "allAnnotatedVariants" and "allCuratedGenes" annotations downloaded March 2019[11]. Specifically, "Cancer genes" were defined as all genes listed in "allCuratedGenes". For SNVs and INDELs "Cancer genes" mutations were defined as either; exact variant matching to "allAnnotatedVariants" or protein truncating variants (PTVs) in genes labeled tumor suppressor genes (TSGs) in "allCuratedGenes".

7. Fusion driver review

BRASS SV results were manually reviewed in IGV[12] if the following criteria were met.

i. readpair.count > 6.
ii. fusion_flag == 0 or fusion_flag > 800.
iii. Either the 5' or 3' gene was "known".

We classified a known fusion gene as the union of ;

- Archer FusionPlex Pan-Heme panel fusion gene targets (n=70) (https://archerdx.com/research-products/blood-cancer-research/fusionplex-pan-heme/)
- New fusions reviewed by Lilljebjörn and Fioretos 2017 [13]: *MEF2D*, *DUX4* & *ZNF384*.
- MLPA (P355) genes: *EBF1*, *IKZF1*, *JAK2*, *CDKN2A*, *CDKN2B*, *PAX5*, *ETV6*, *BTG1* & *RB1*.
- Ph-like fusions described in Roberts et al. 2017 [14]: *ABL1*, *ABL2*, *CSF1R*, *PDGFRA*, *PDGFRB*, *EPOR*, *JAK2*, *TYK2*, *FLT3*, *NTRK3*, *PTK2B*, *BLNK*, *CBL*, *P2RY8* & *CRLF2*.

- *CDX2/UBTF::ATXN7L3* as defined by Kimura et al. 2022[15]: *FLT3*, *PAN3*, *UBTF* & *ATXN7L3*.

9. IGH enhancer hijacks

IGH enhancer hijacks are rearrangements which are oncogenic by the somatic proximity of a gene to the IGH loci. Therefore, to supplement the standard fusion calling by BRASS, we imported the BRASS calls into gGnome (https://github.com/mskilab/gGnome) which constructs the SV calls into a graph. For each graph the gGnome ``proximity" function was used to "walk" from the Eμ *IGH* super enhancer locus to genes in GENCODE v29[16,17]. Candidate IGH walks <100 kbp were annotated using the Atlas of Genetics and Cytogenetics in Oncology and Haematology and Mitelman Database of Chromosome Aberrations and Gene Fusions in Cancer[18,19].

10. DUX4 rearrangement detection

The *IGH::DUX4* enhancer hijack is particularly difficult to identify as *DUX4* resides in D4Z4 repeat arrays which occur at multiple loci (chr4, chr10 & chrUn_gl000228) across the genome (GRCh37) and therefore, reads mapping across the translocation-defining *IGH::DUX4* will be filtered due to low mapping quality. To solve this, we used the GRIDSS SV caller (run at MSKCC on the Isabl platform as described by Shukla et al. 2022[20]), which takes an assembly approach to candidate SV. This allows to identify single breakends (SV unambiguously anchored at only one locus).

For each single breakend we used BLAT to map the unplaced sequence to hg19. BLAT was performed using gfServer/gfClient (v36). This was run as described

(https://genome.ucsc.edu/FAQ/FAQblat.html#blat11) over the sequence contained in the ALT column of the GRIDSS VCF for single breakends anchored in the IGH locus. BLAT results were then processed by pslReps (https://genome.ucsc.edu/goldenPath/help/blatSpec.html#pslRepsUsage) and pslScore.pl (https://genome.ucsc.edu/FAQ/FAQblat.html#blat4) to approximate web-based results given by https://genome.ucsc.edu/cgi-bin/hgBlat. Single breakend sequences which mapped to chrUn_gl000228 were reviewed further in IGV[12].

11. Coding driver review

One of the most powerful variant filters in WGS is the so-called "panel of normals" (PON), that removes recurrent mapping and technical artifacts. These panels are usually made from blood DNA, so true hematological variants have the potential to be falsely filtered out. Then, we focussed on recovering any mutations for genetic subtypes with coding drivers (*PAX5* and *ZEB2*). For *PAX5* and *ZEB2*, SNVs which were only flagged by CaVEMan with the "VUM" (PON) filter were manually reviewed. These recovered PAX5 P80R from the standard WSI pipeline filters.

12. Ploidy subtype review

In this cohort our objective was to identify cases to review for ALL ploidy subtypes: hypodiploidy (chromosomes < 40) and hyperdiploidy (chromosomes > 50).

We used the ASCAT ploidy estimate (divided by 2 so that normal ploidy = 1) to identify candidate ploidy subtypes, within cases with optimal purity/ploidy fits as described above.

To identify candidate ploidy subtypes for manual review, ASCAT ploidy/2 threshold were set to.

- Hypodiploidy
    - ASCAT ploidy < 0.87 (40/46)
- Hyperdiploidy
    - ASCAT ploidy > 1.09 (50/46)

Additionally we determined arm-level events using ASCAT copy data, arm-level events were called when the sum of CNA (loss or gain) was > 70% of the chromosome arm length.

13. Germline review

Normal samples were processed for germline variation using the Isabl platform at MSKCC as described in Shukla et al 2022[20]. Mutations were assessed in genes previously associated with ALL predisposition (*ETV6*, *IKZF1*, *PAX5* & *TP53*) and variants that were called by at least 1 caller and with a max allele frequency in Genome Aggregation Database (gnomAD) < 1% were reviewed for evidence in ClinVar[21,22].

14. Single base substitutions (SBS), mutation signature analysis

Mutation signature analysis was performed using the R/Bioconductor "MutationalPatterns" (v3.5.6) package[23] using the lymphocytes-derived 7 signature of SBS mutational catalog from Machedo et al. 2020 as reference[24,25] and the "fit_to_signatures_strict" function using max_delta = 0.01 and method = "best_subset". Additionally, comparison of PD40812 to the thio-dMMR reference signature[26] was performed using the "plot_compare_profiles" function. SNVs from CaVEMan were filtered to provide high-quality input as described above.

15. RAG mediated deletion identification

To identify RAG mediated deletions, we took 20bp flanking sequence upstream and downstream from each assembled BRASS deletion (i.e. with exact defined breakpoint). These sequences were formatted to FASTA and submitted to a local installation of MEME (5.0.5) (http://meme-suite.org/meme-software/5.0.5/meme-5.0.5.tar.gz), using the following command:

"meme <input.fasta> -dna -mod zoops -revcomp -nmotifs 15 -minw 6 -brief 5000 -p 5 -oc <output>".

The arguments were set to the website defaults except the maximum number of motifs to report, which was increased from 3 to 15, as per Papaemmanuil et al. 2014[27].

16. Telomere length

Telomere length was estimated individually across tumor and normal samples using TelomereCat[28]. Each WGS BAM was processed to identify telomere reads using "bam2telbam", then telomere length was estimated from the resulting "TELBAM" using "telbam2length" with the following arguments "-p 4 -v 2 -N 100".

RNA-seq library preparation, sequencing and alignment

1. Library preparation and sequencing

RNA-seq was performed using stranded oligo(dT) pulldown to a target coverage of 50 million reads (2x 75bp paired reads on Illumina HiSeq 4000 system).

2. Alignment

RNA sequence files were aligned to GRCh37d5 using STAR (version 2.5.0c), with Ensembl release 75 gene data[29].

RNA-seq analysis (WSI)

1. Post-alignment QC

Sequencing coverage estimates were estimated by the sum of mapped read pairs length in bases divided by an exonic target interval covering 96,435,807 bases (Supplemental Table 4).

After alignment we used Somalier[4] to confirm sample identity between WGS tumor normal pairs and RNA-seq data (Supplemental Table 7). Somalier identified three RNA-seq samples with > 0.5 relatedness (PR43260c, PR43262c & PR37187c), suggesting possible cross contamination.

2. RNA classification

To provide robust RNA classification we used the following 4 classifiers:

1. ALLSorts [30]
2. ALLSpice [31]
3. ALLCatchR (https://github.com/ThomasBeder/ALLCatchR)
4. MD-ALL tool set (https://github.com/gu-lab20/MD-ALL)
    a. PhenoGraph
    b. K nearest neighbors

Appropriate input data was formatted from gene counts from HTSeq (v0.7.2) over Ensembl release 75 gene data (Homo_sapiens.GRCh37.75.gtf.gz). ALLSorts and ALLCatchR were run using default commands, ALLSpice was run without the optional sex and age covariates.

The MD-ALL tool suite is based on clustering a sample with unknown subtype to a reference dataset (n=2042). As we did not have a complete overlap of genes between our study and the COH subtype prediction gene set (1664/2251 genes from "boruta_gene"), we tuned the parameters for the two prediction tools by minimizing prediction error for the reference dataset.

We were able to eliminate reference set prediction error for PhenoGraph by setting k to 11 using all 1664 genes and for KNN (k=5) by performing the initial tSNE using all 1664 genes with a perplexity of 45. We then performed PhenoGraph and KNN subtype prediction individually using these optimal parameters, using the following steps:

1. Normalize single sample count
2. Add to COH reference (n=2042)

3. Predict subtype
    c. PhenoGraph - Individual (1664 genes, K = 11)
    d. KNN (k=5) - Individual (tSNE - 1664 genes, perplexity 45)

As we were concerned by any residual batch effects between our study cohort RNA data and the COH reference data set, we also ran both tools using all study samples using the following workflow:

1. Combine single sample normalized counts (n=33)
2. Add all 33 normalized sample counts to COH reference (n=2042)
3. Predict subtype
    a. PhenoGraph - Cohort (1664 genes, K = 11)
        i. All 33 samples have subtypes predicted from the combined count data (n=2075)
    b. KNN (k=5) - Cohort (tSNE - 1664 genes, perplexity 45)
        i. Generate initial tSNE from combined count data (n=2075)
        ii. For each study sample, predict subtype individually by subsetting the tSNE sample to the sample to be predicted and all COH reference samples (n=2043)

3. RNA classifier consensus

To identify RNA subtype consensus we used the subtype consensus of any two of the trained classifiers (ALLSorts, ALLSpice and ALLCatchR) and compared this to a consensus of the PhenoGraph and KNN individual and cohort subtype predictions using the COH reference data to finalize the RNA classification.

4. Sample QC

We also sought to ascertain which cases may have low diagnostic potential due to either.

3. Cross contamination identified by Somalier (relatedness > 0.5)
4. Inferred low purity tumor
    a. ALLSpice tissue inference was not “B-cell ALL”
    b. Outlier ALLCatchR blast %

This was coded as “High” or “Low” in the “RNA sample quality” column in Supplemental Table 11 & Supplemental Table 13.

RNA-seq analysis (MSKCC)

RNA sequencing data was also processed within the Isabl platform at MSKCC as described in Shukla et al 2022[20].To identify gene fusion, we used two different callers:

1. FusionCatcher (v1.0.0, https://github.com/ndaniel/fusioncatcher)
2. CICERO (v1.9.2 , https://github.com/stjude/CICERO)

We combined the results with the “get_BALL_fusion” function from the MD-ALL R package (https://github.com/gu-lab20/MD-ALL), which filters and annotates known B-ALL fusions and partner genes for manual review.

SoC comparisons

1. MLPA

The MLPA P335 assay is comprised of 8 gene centric copy number assays (covering *EBF1*, *IKZF1*, *CDKN2A*, *CDKN2B*, *PAX5*, *ETV6*, *BTG1* & *RB1*) and 5 genes on the chromosome X PAR1 region (*SHOX*, *CRLF2*, *CSF2RA*, *IL3RA* & *P2RY8*) to identify the *CRLF2::P2RY8* fusions from the loss of the interstitial genes *CSF2RA* and *IL3RA*. MLPA event calls for 51/52 samples were available from Moorman et al. 2022, called as previously described by Schwab et al 2010[1,2]. For WGS, BRASS SV events and copy number calls from ASCAT were annotated as loss, normal or gain for comparison to MLPA results calls as well as the exons covered by these events using MAME select transcripts[32] as reference (Supplemental Table 5).

a. Comparison sample QC

To standardize the comparison between MLPA and WGS we removed three samples;

1. PD37198 due to extremely low WGS purity (~13%).
2. PD40813 where the MLPA had likely been performed on a low purity sample with no event by MLPA but with high WGS purity (89%) and multiple MLPA target events.
3. PD40823, a case with high probe variance in the MLPA assay.

These resulted in 48/52 samples available for comparison.

Tables

In supplementary xlsx

Supplemental Table 1: Demographic and clinical characteristics of cohort

Supplemental Table 2: Sample source

Normal DNA Sample source (replacement) describes the samples selected to replace the buccal samples which failed. MRD data pertains to the final Normal sample sequenced.

Supplemental Table 3: WGS QC metrics

Mapping metrics for WGS data, Sequencing coverage is defined as the sum of mapped read pairs length in bases divided by the aligned reference in bases (GRCh37d5 3,137,282,682)

Supplemental Table 4: RNA QC metrics

Mapping metrics for RNA data, Sequencing coverage is defined as the sum of mapped read pairs length in bases divided by an exonic target interval covering 96,435,807 bases.

Supplemental Table 5: Transcripts used for MLPA comparison

MLPA target gene transcripts from MAME select (https://www.ncbi.nlm.nih.gov/refseq/MANE/) for comparison to WGS.

Supplemental Table 6 Five samples removed due to low SNV burden

Supplemental Table 7: Post QC study cohort

Supplemental Table 8: Driver allocation

Supplemental Table 9: 6 B-other cases

Supplemental Table 10: SV events (BRASS) called within KDM6A in PD40803

Supplemental Table 11: Consensus RNA subtype classification (n=33)

Supplemental Table 12. RNA fusion results (n=33)

Supplemental Table 13: Comparison of WGS and RNA subtype classification (n=31)

Supplemental Table 14: DUX4 rearrangements details

Details of GRIDSS rearrangements supporting DUX4r, "IGH::DUX4 event type" describes the relationship of the IGH locus to DUX4 containing sequence. The 2 cases where IGH anchored events mapped to telomere repeats are labeled "IGH-telomere" .

Supplemental Table 15: IGH enhancer hijacks

Supplemental Table 16: Additional deletions called by WGS over MLPA

Supplemental Figures

Supplemental Figure 1: Study cohort sample selection. CONSORT diagram detailing 652 B-cell precursor Acute Lymphoblastic Leukemia (BCP-ALL), of which 394 B-ALL cases met the sample requirements, 221 were classed as B-Other using standard of care (SoC) cytogenetics. These were further screened with the additional research FISH panel and MLPA (P335) to enrich for non Ph-like cases, this yielded 89 cases eligible for sequencing of which 58 cases had available samples.

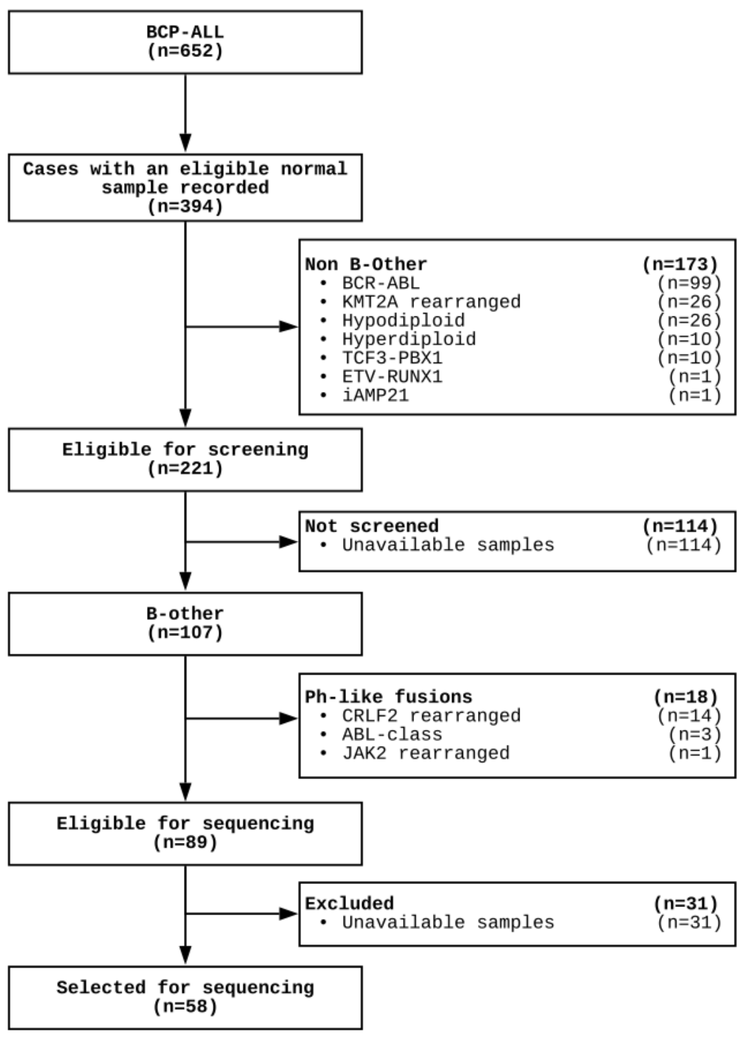

Supplemental Figure 2: WGS coverage by library type for tumor a) and normal b) showing no significant difference in coverage when tested with a Mann-Whitney U-test

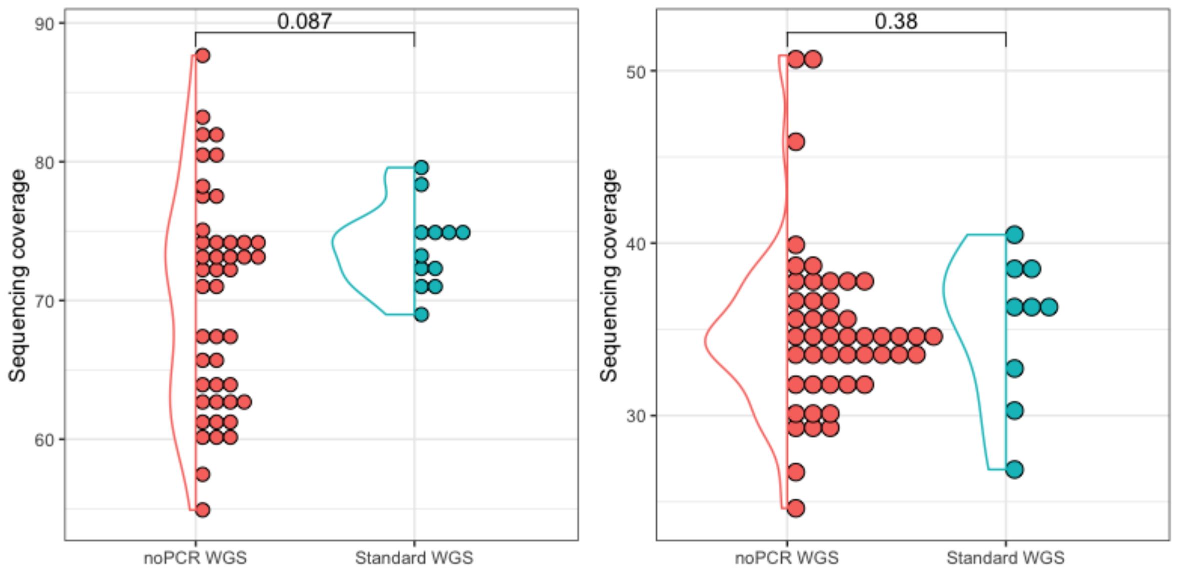

Supplemental Figure 3: SBS mutational catalog

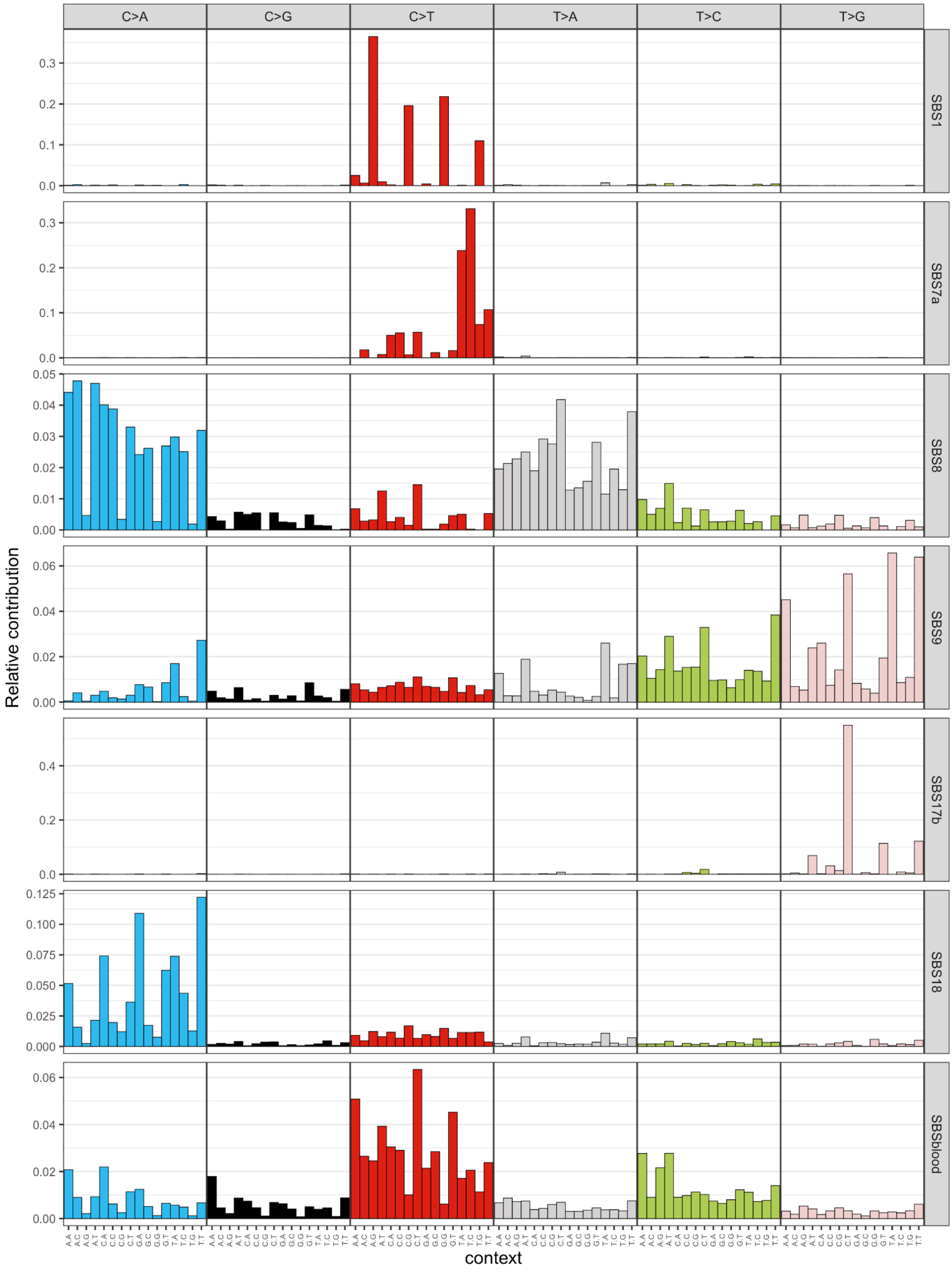

Legend: 96 context plots of SBS mutational catalog used for fitting from Machado et al. 2020

Supplemental Figure 4: SNV burden by purity adjusted tumor read depth

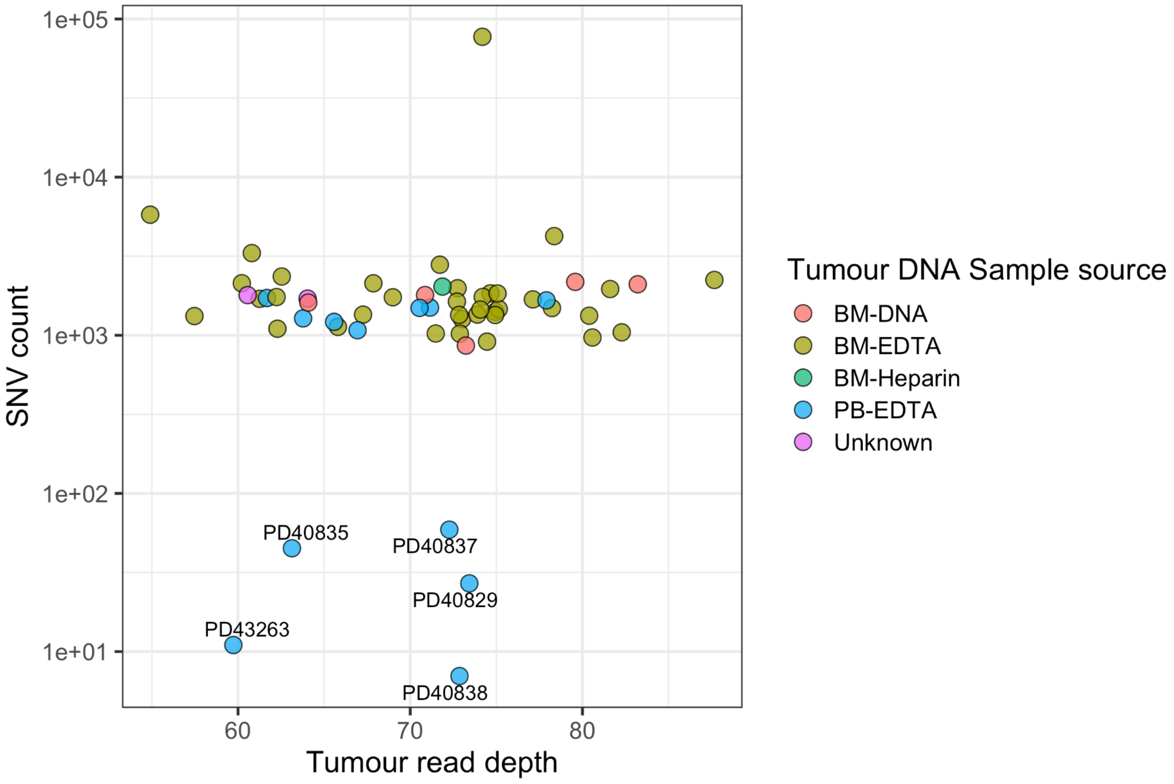

Legend: Plot of total SNV burden by tumor readdepth shows 5 low SNV burden outliers (<100) all from peripheral blood.

Supplemental Figure 5: ASCAT copy number profiles of 5 cases with low (<100) SNV burden

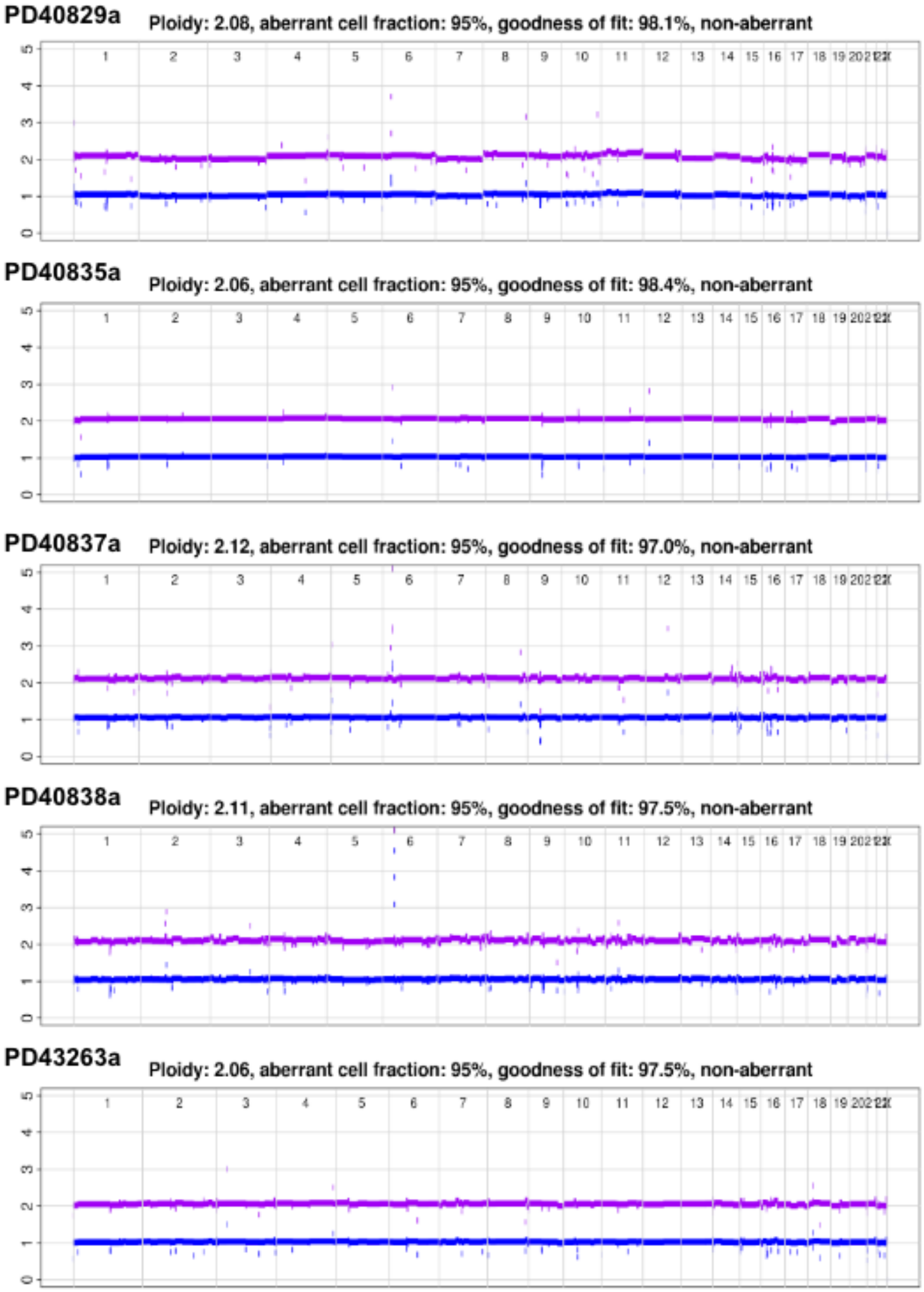

Legend: ASCAT ploidy plots (total copy number in purple, minor copy number in blue) of 5 cases removed due to low (<100) SNV burden.

Supplemental Figure 6: Oncoplot of 47 B-other cases

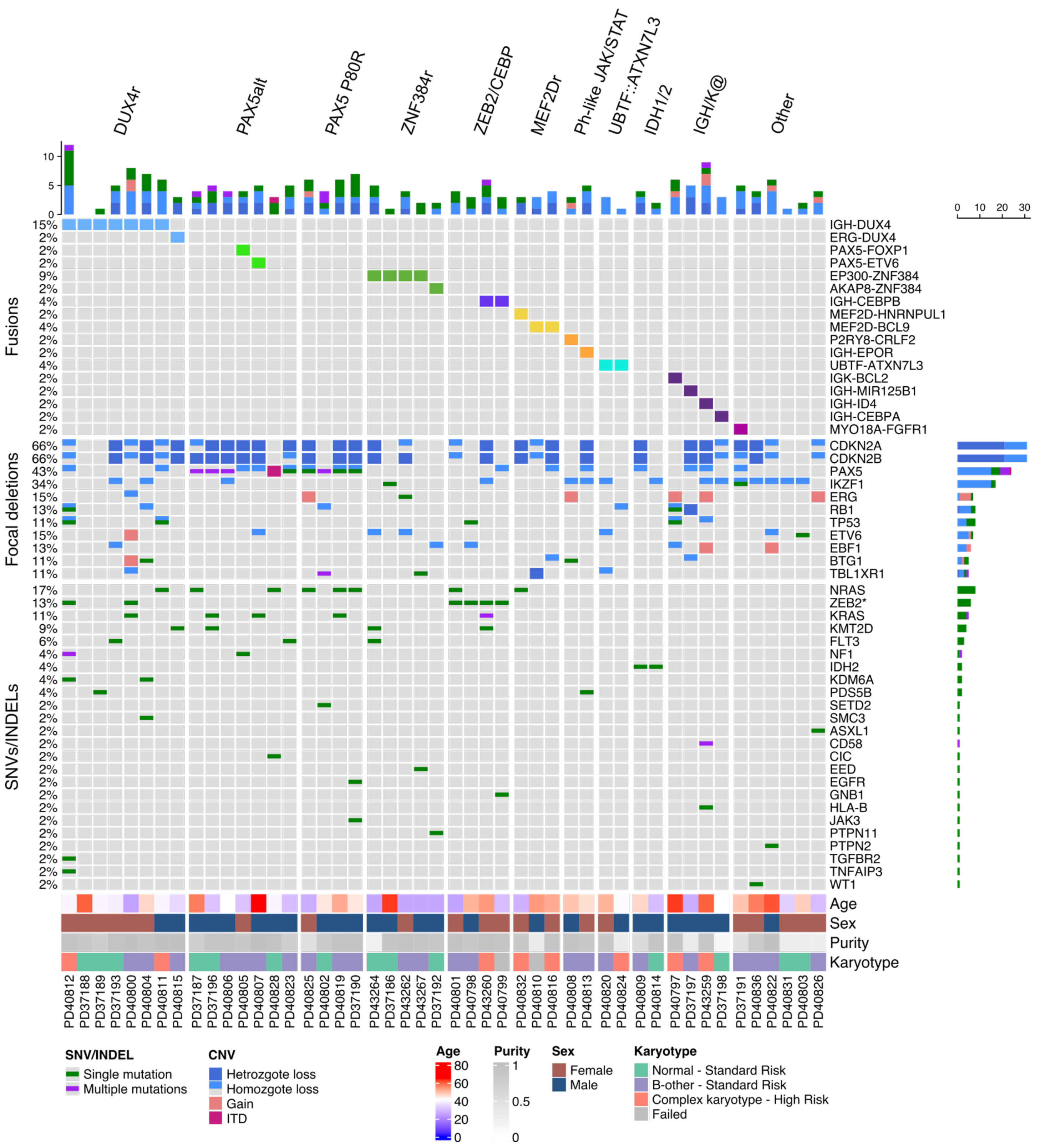

Legend: Oncoplot of 47 B-Other cases, upper panel shows driver fusions, middle panel genes with recurrent focal deletions and lower panel with recurrently mutated genes in OncoKB™ or ZEB2 (marked with asterisk).

Supplemental Figure 7. RNA sample purity inference

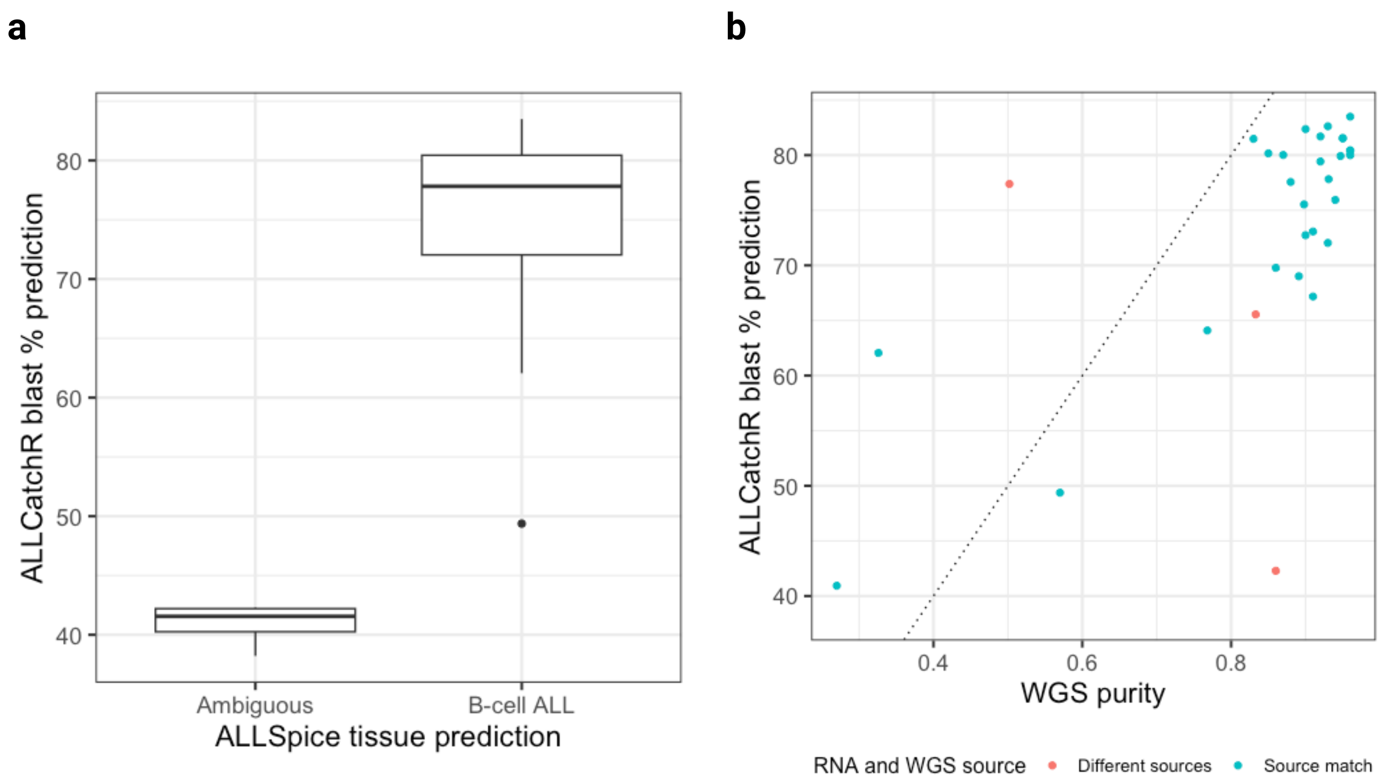

Legend: Plot a) shows the relationship between ALLSpice tissue prediction "Ambiguous" (n=4) with lower Blast % inference by ALLCatchR in 33 RNA samples. Plot b) shows the correlation between WGS inferred purity against ALLCatchR Blast % in 31 cases with WGS purity. This shows good correlation between RNA and WGS samples with matching sources, with the one low WGS and high ALLCatchR Blast % outlier (PD37194) was classified as High hyperdiploid therefore likely accounting for the discordance.

Supplemental Figure 8: Chr17 copy number and SV profiles of 4 cases with TP53 mutations

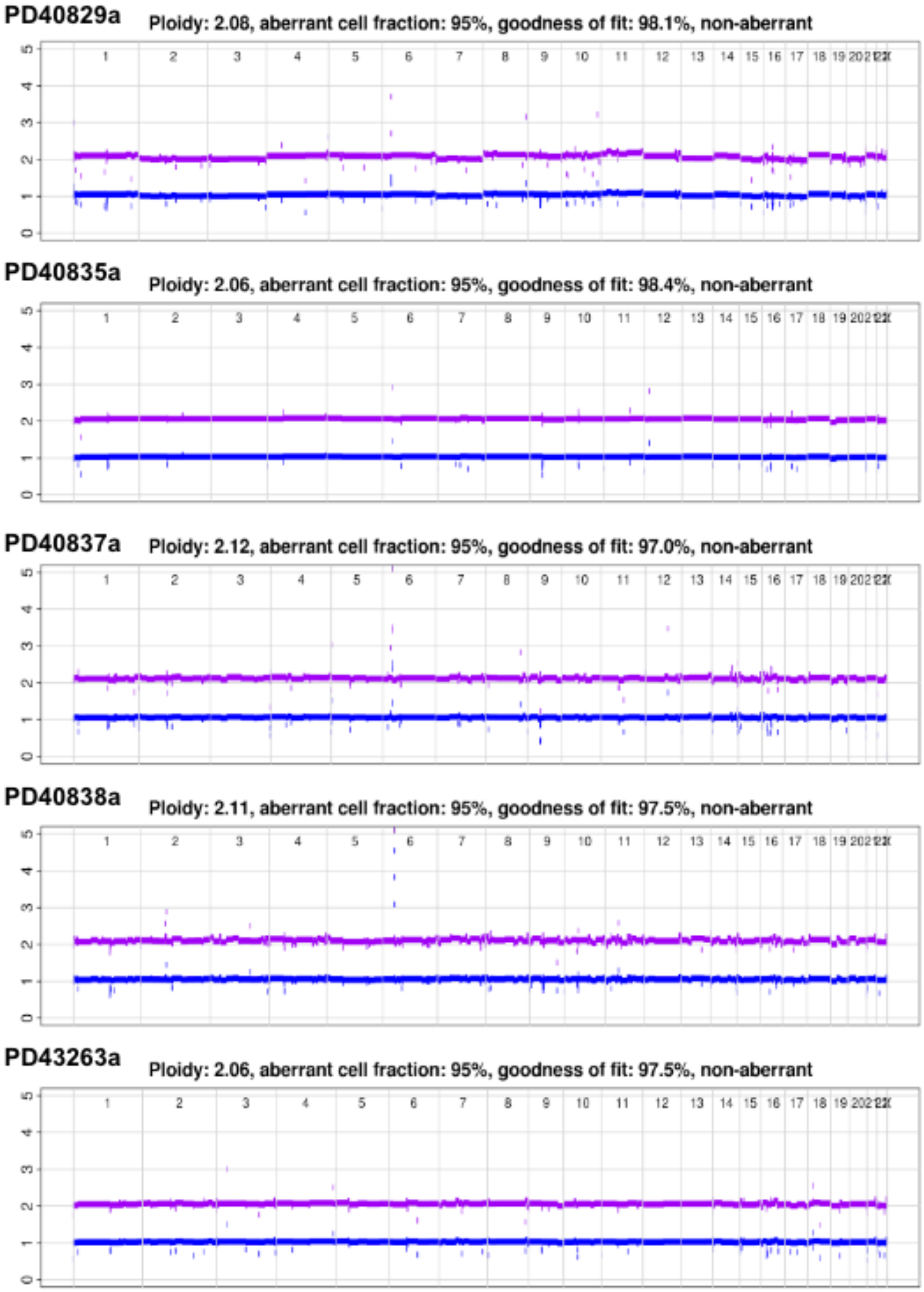

Legend: Integrated SV and copy number plots for chr17 for cases with TP53 mutations. For each plot the coloured arcs denote SVs: inversions on the top horizontal axis (head to head and tail to tail) and tandem duplication and deletion on the lower horizontal axis.

Supplemental Figure 9. Read depth profile over ERG and IGH loci in 8 DUX4-r cases

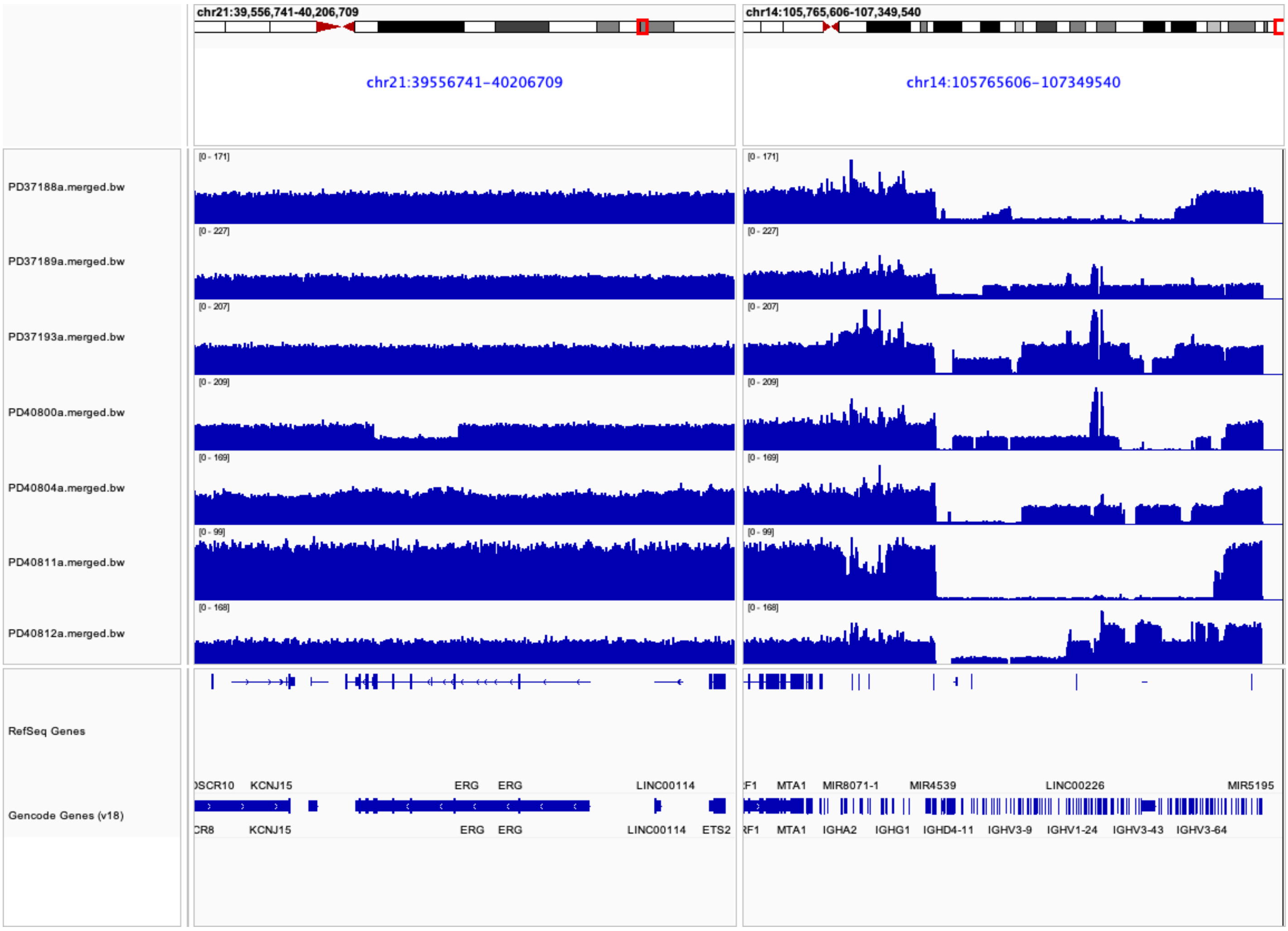

Legend: IGV plot showing read coverage plot for DUX4-r cases at ERG (LHS) and IGH loci (RHS), showing only a single case with ERG deletion, while clonal IGH rearrangements are observed across all cases.

Figure 10: Swimplot of B-other subtypes (n=47)

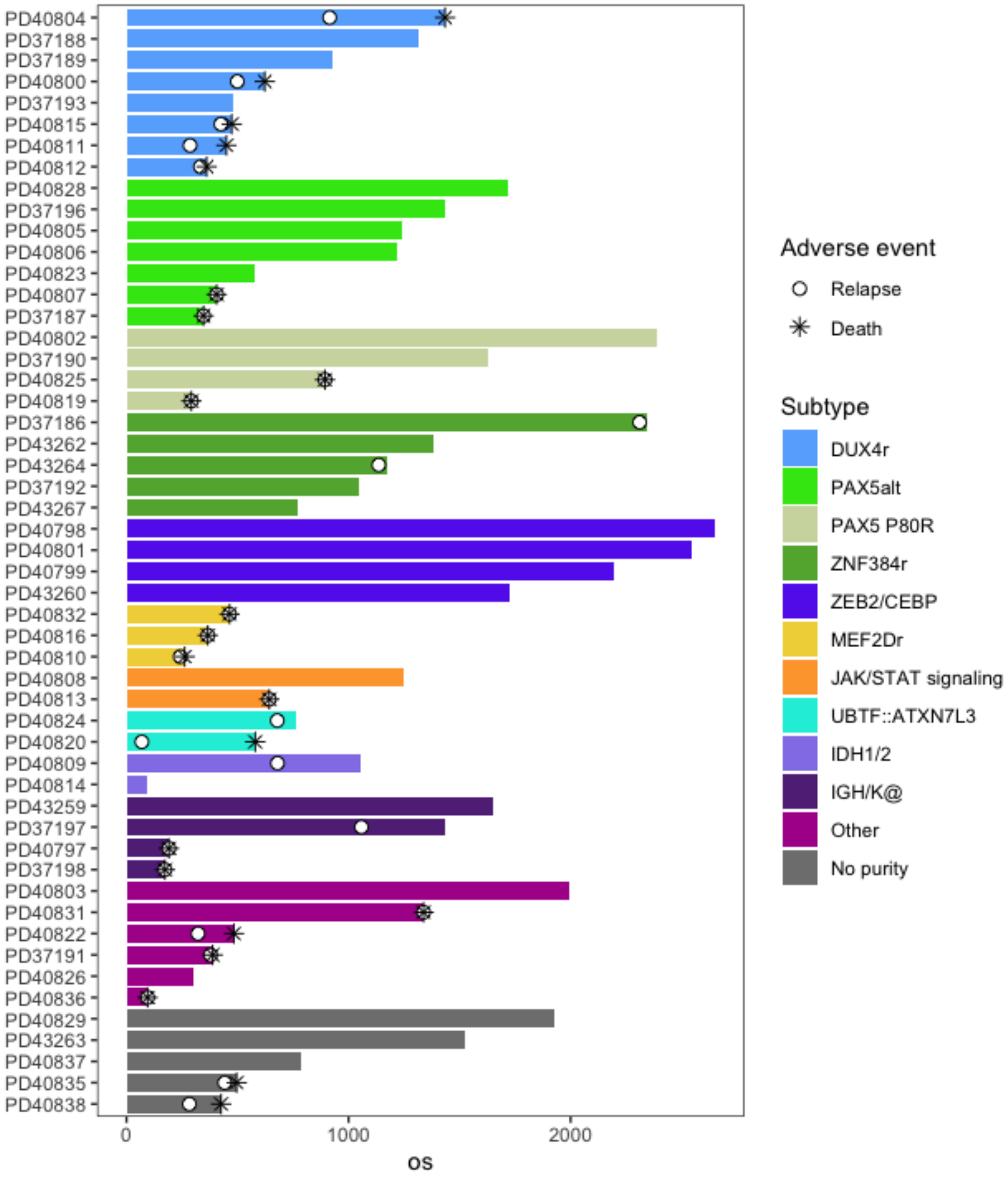

Legend: Swimplot of B-other subtypes (n=47: Swimplot showing overall survival and annotated for event (relapse and death).

Figure 11: Biallelic targeting of *PAX5* within PAX P80R and PAX5alt subtypes

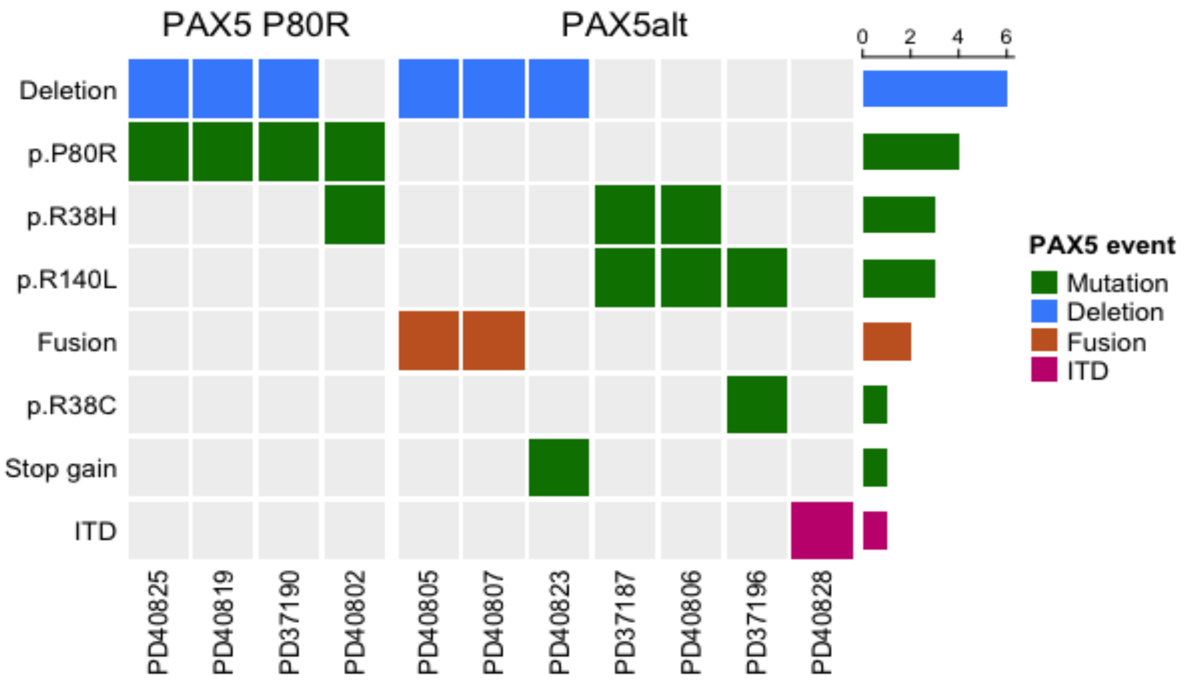

Legend: Tile plot showing biallelic targeting of *PAX5* by mutations and LOH across PAX P80R and PAX5alt subtypes. Deletion - denotes any deletion event over *PAX5*, ITD - Internal tandem duplication.

Supplemental Figure 12: Diverse PAX5alt genotypes; *PAX5* ITD, *PAX5* stopgain and *PAX5* LOH

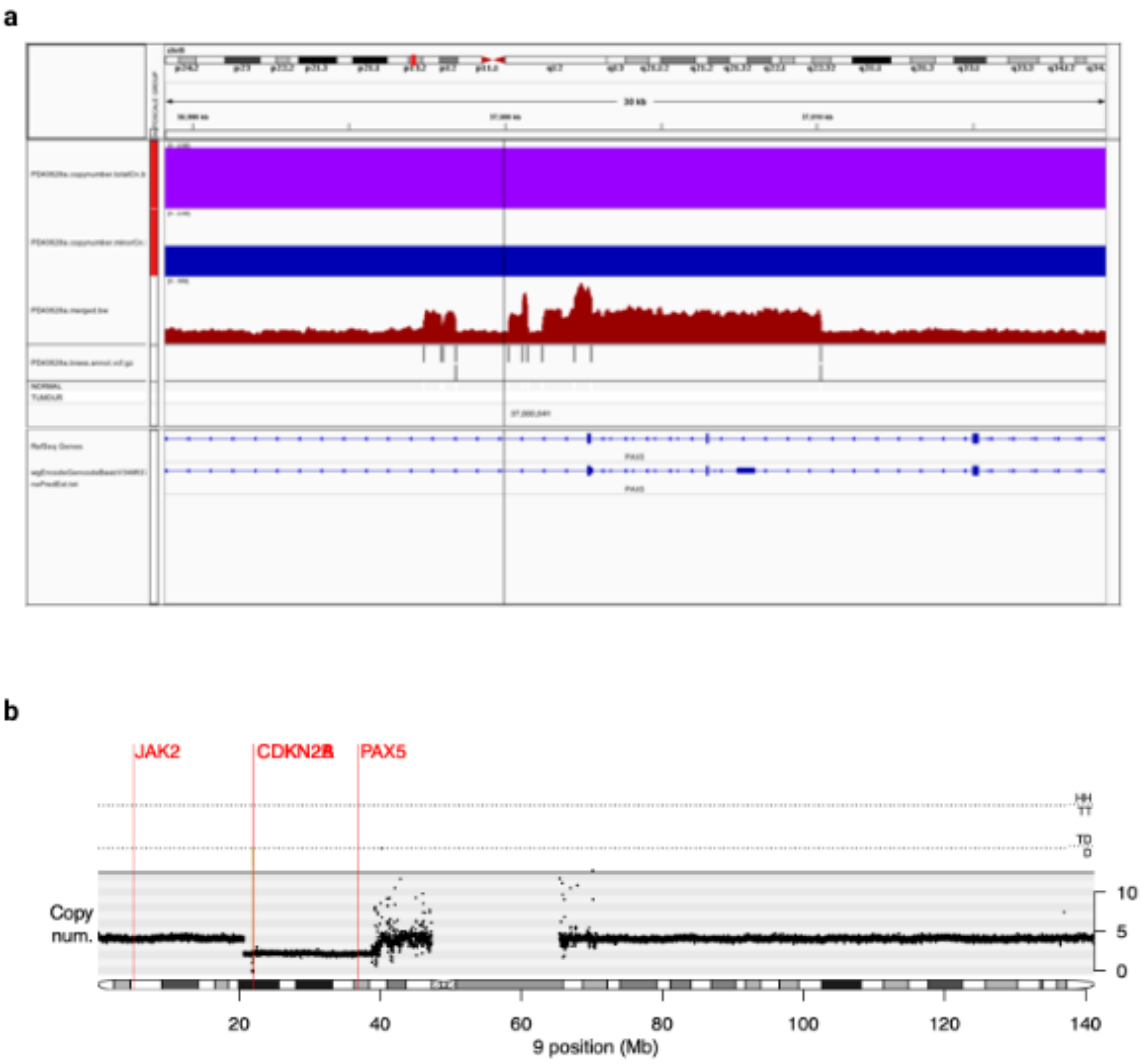

Legend: Diverse PAX5alt genotypes; a) IGV view of *PAX5* ITD (PD40828), showing; No copy change called by ASCAT (total copy number in purple, minor copy number in blue), WGS read coverage (red track) showing copy change over exons 5 and 4, along with extensive SVs called by BRASS on the track below. b) Integrated SV and copy number plot for chr9 showing the LOH of PAX5.p.M335fs*68 from a sub arm loss on chr9p in for PD40823.

Supplemental Figure 13: PAX5 p.P80R chr9 copy number profile.

PD40819a chr9 PAX5 p.P80R LOH

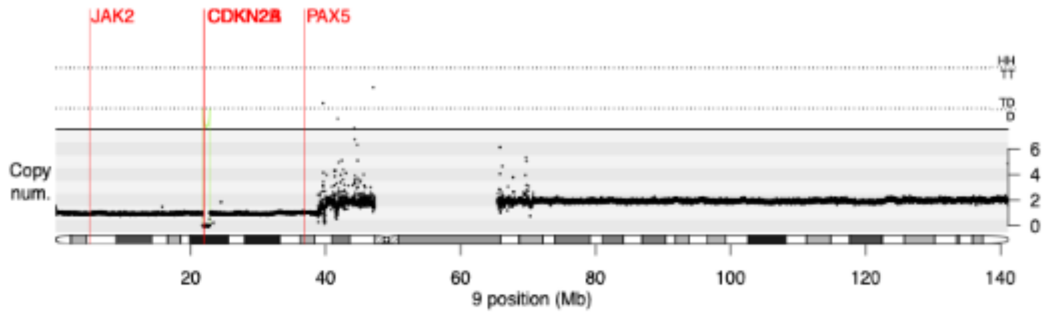

PD40825a chr9 PAX5 p.P80R LOH

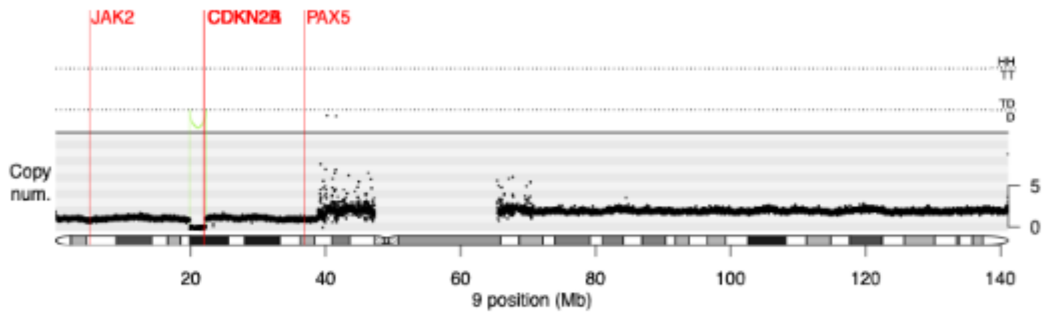

PD37190a chr9 PAX5 p.P80R LOH

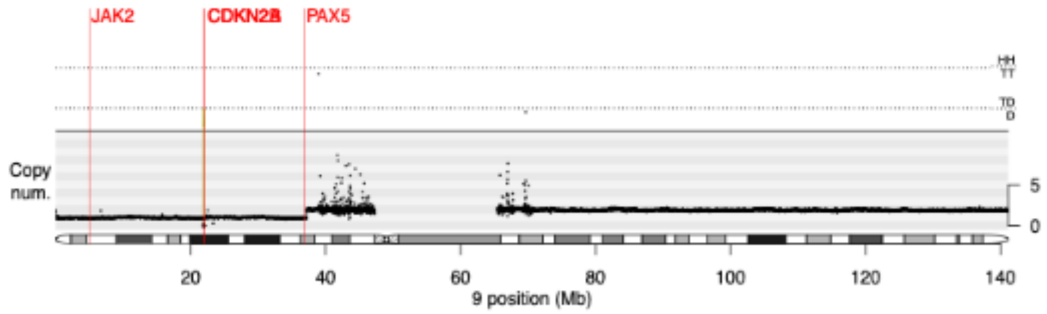

PD40802a chr9 PAX5 p.P80R & p.R38H

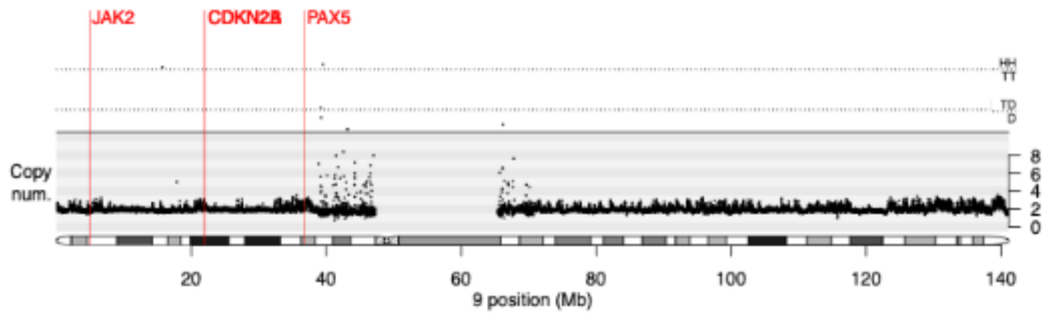

Legend: Integrated SV and copy number plots for chr9 for PAX5 P80R cases. For each plot the coloured arcs denote SVs: inversions on the top horizontal axis (head to head and tail to tail) and tandem duplication and deletion on the lower horizontal axis. Gene of interest (*JAK2*, *CDKN2A*/*B*, *PAX5*) are marked by a red vertical line.

Supplemental Figure 14: Consistent copy number gain over *MEF2D* locus in *MEF2D*-r cases.

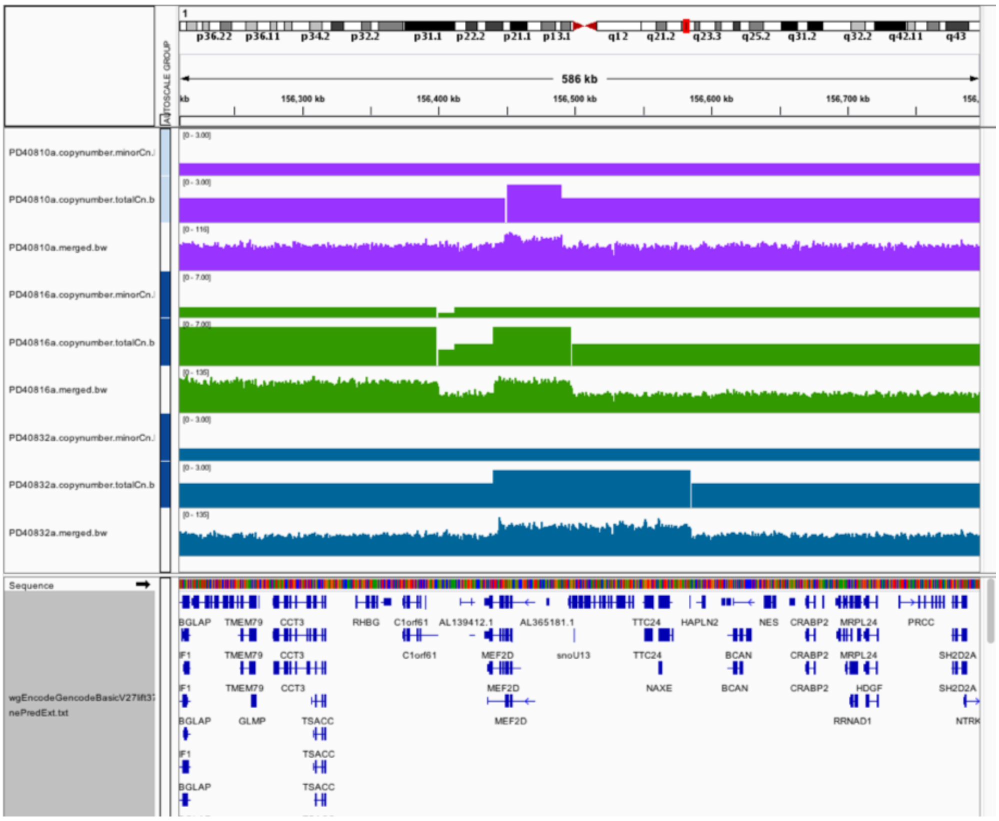

Legend: IGV view of ASCAT total copy number over *MEF2D* locus, showing recurrent copy gain in each *MEF2D*-r cases.

Supplemental Figure 15: Concurrent FLT3/PAN3 microdeletion in *UBTF::ATXN7L3* cases

a

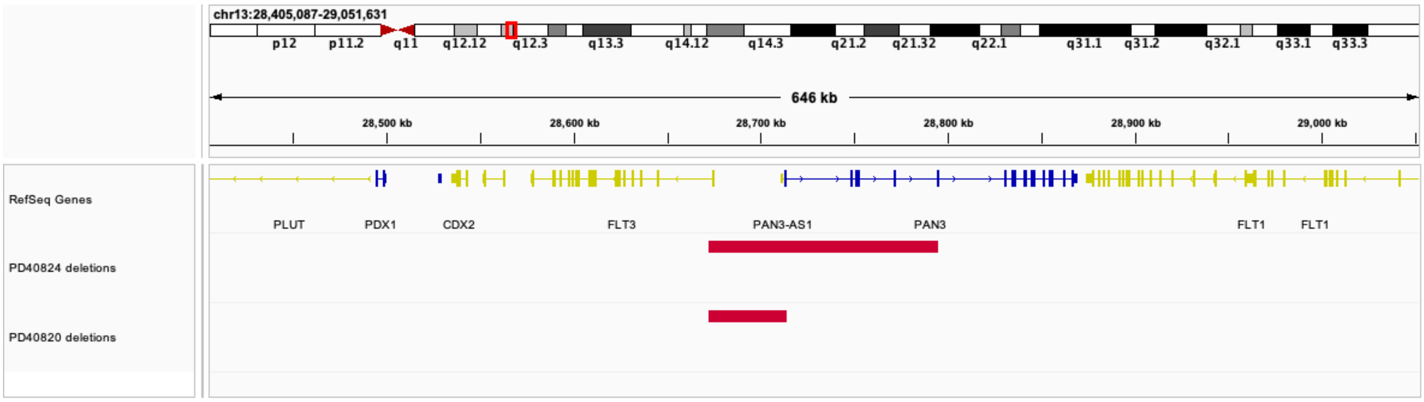

b

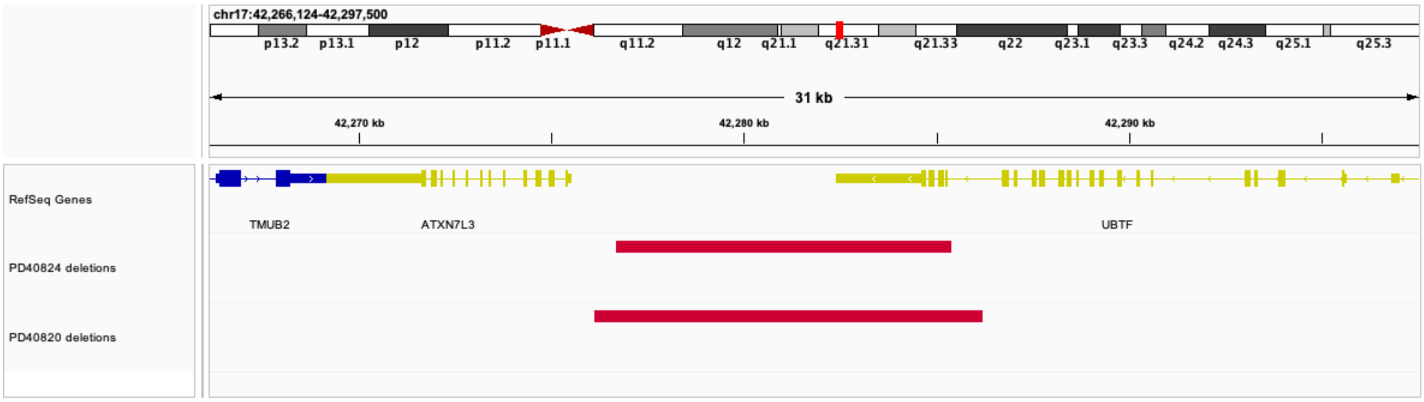

Legend: IGV locus plots in two *UBTF::ATXN7L3* cases showing a) concomitant deletions across exon 1 of FLT3 and into PAN3 and b) *UBTF::ATXN7L3* fusions. Deletion shows in red called by BRASS, gene track colour denotes strand (blue = forward, gold = reverse).

Supplemental Figure 16: Clonal IDH2 p.R140Q in two cases

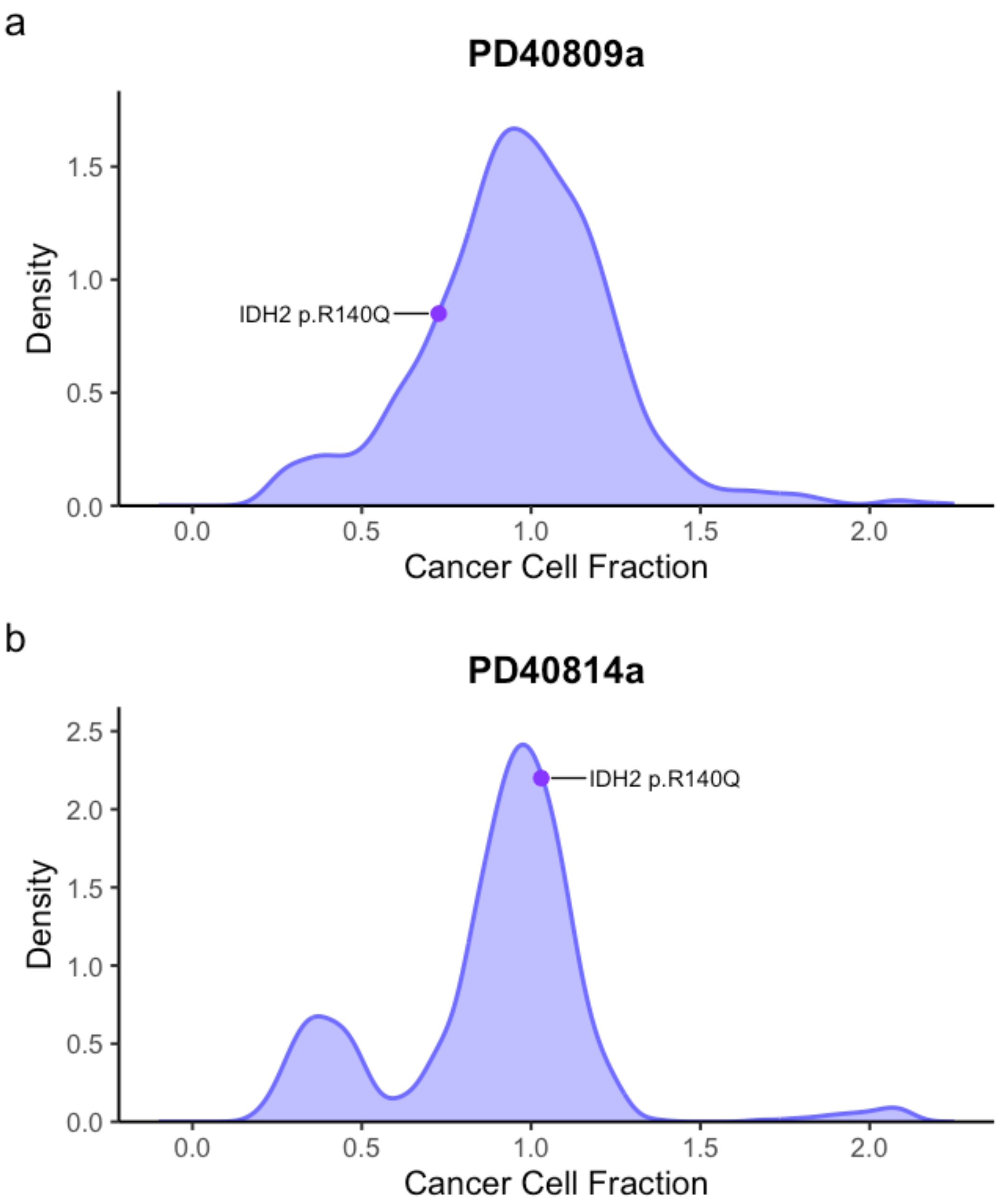

Legend: Density plots of cancer cell fraction (copy adjusted SNV VAF), showing clonal nature of IDH2 R140Q variant in a) PD40809a and b) PD40814a.

Supplemental Figure 17: ERG::DUX4 with ERGalt expression in PD40815

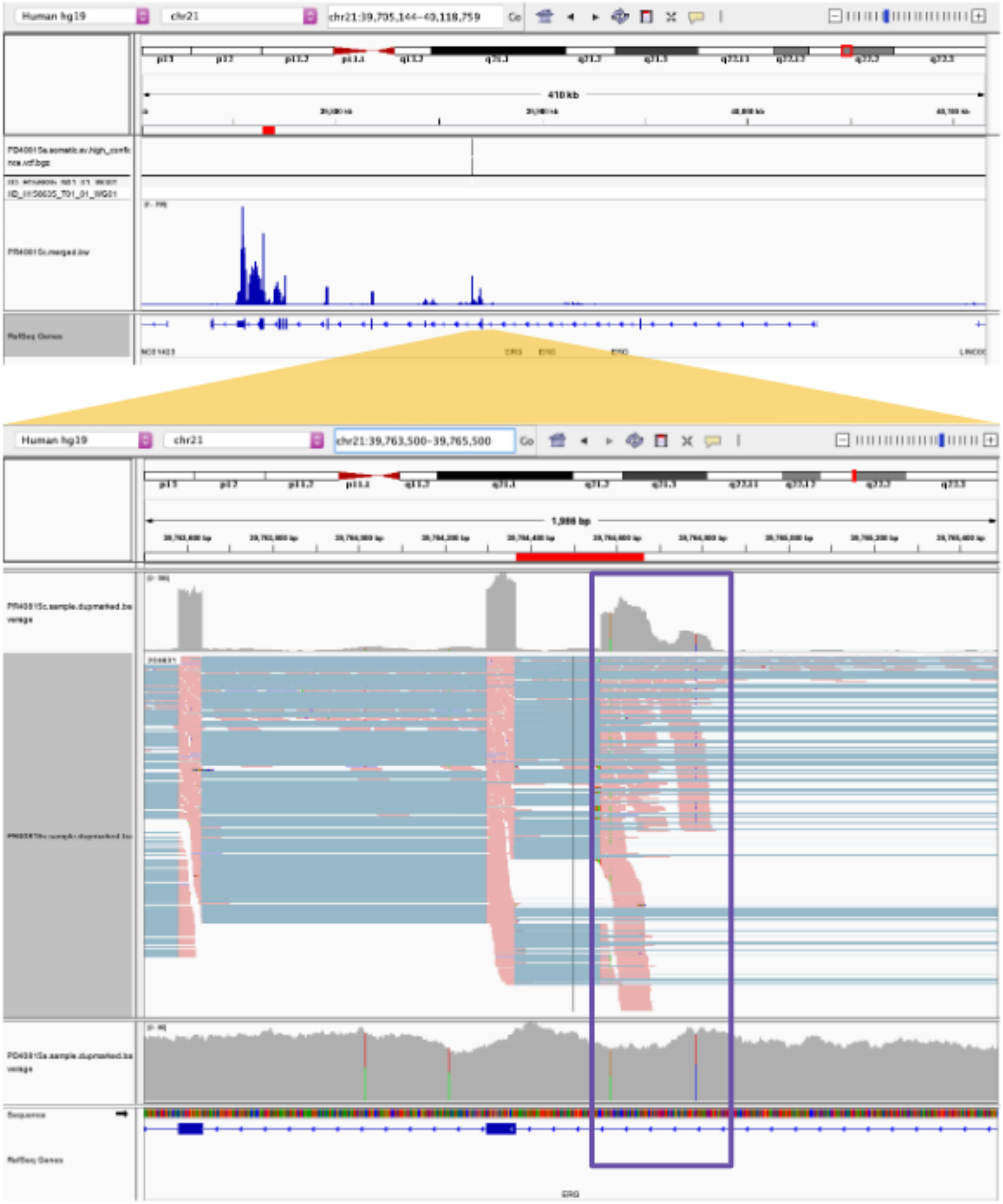

Legend: IGV view of RNA data showing ERGalt expression in PD40815, upper panel showing breakpoints of balanced translocation and RNA read coverage across *ERG* transcript, lower panel zoomed in detail of expression of exon 6 alt (outlined in purple).

Supplemental Figure 18: *IGH::MIR125B1* (PD37197a)

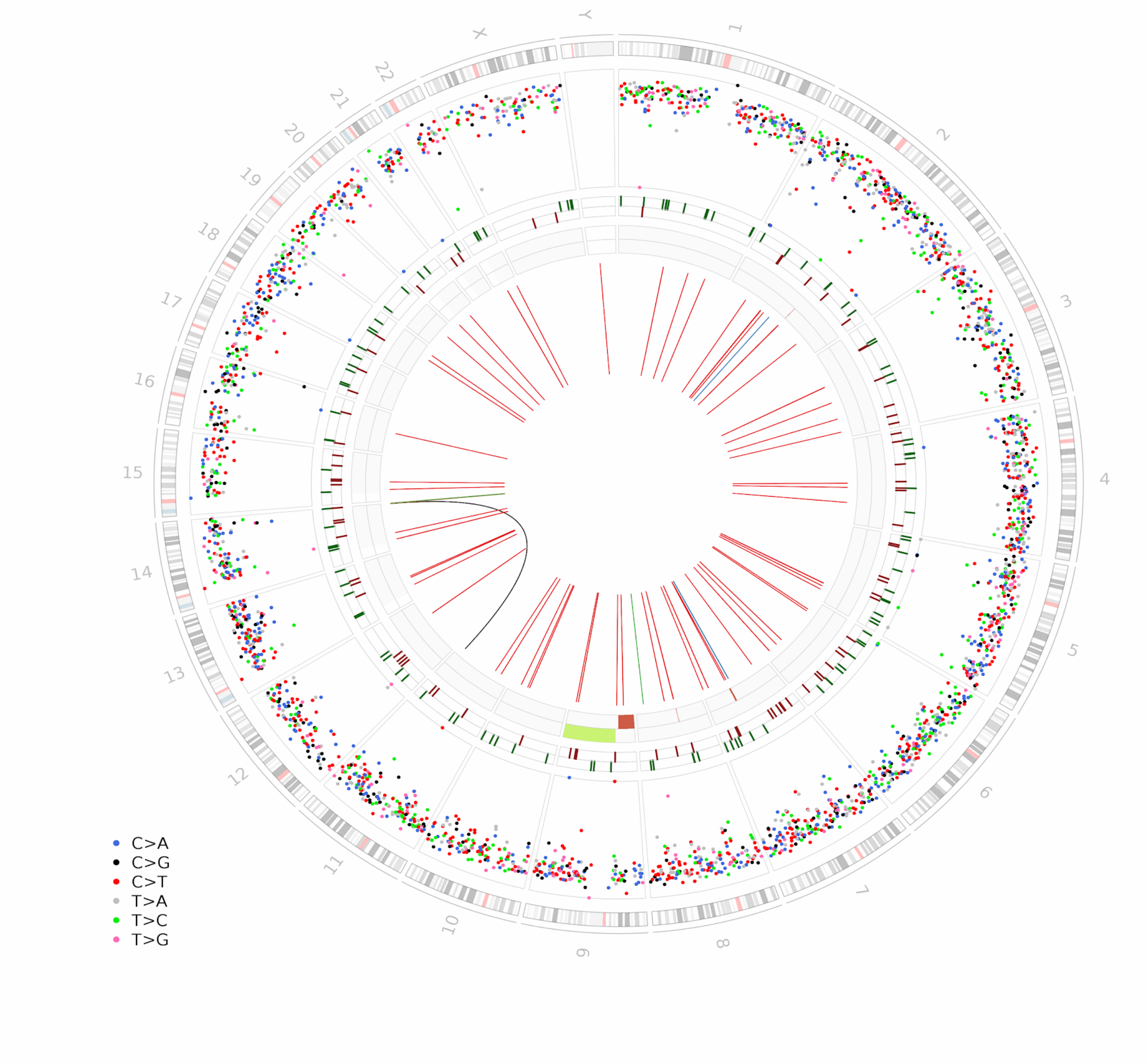

Legend: Circos plot showing the high focal deletion burden of this sample. The outermost ring shows the intermutation distance for all SNVs color-coded by the pyrimidine partner of the mutated base. The middle ring shows small insertions (green) and deletions (red). The innermost ring shows copy number changes, and the arcs show SVs.

Supplemental Figure 19: Relationship between age of diagnosis and estimated SBSblood mutation burden.

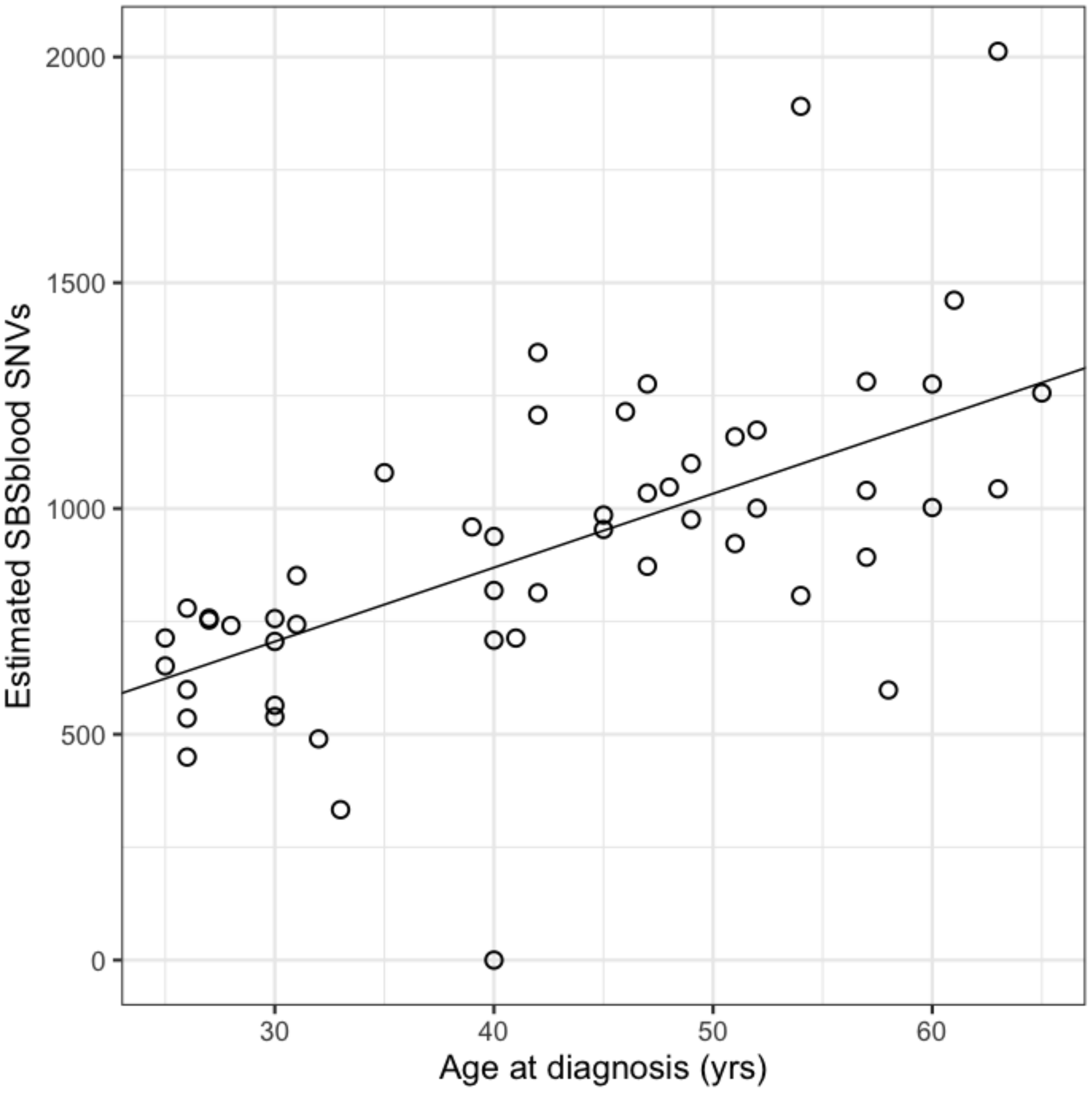

Legend: SBSblood mutation count was estimated by multiplying the SBSblood exposure by total SNV count. Robust linear regression estimated 16.3825 mutations per year (95% CI 11.69416 - 21.07075). The case with 0 SBSblood contribution is PD40812 an *IGH::DUX4* case with SBS1 hypermutation.

Supplemental Figure 20: *IGH::DUX4* hypermutator mutational signature fitting

a

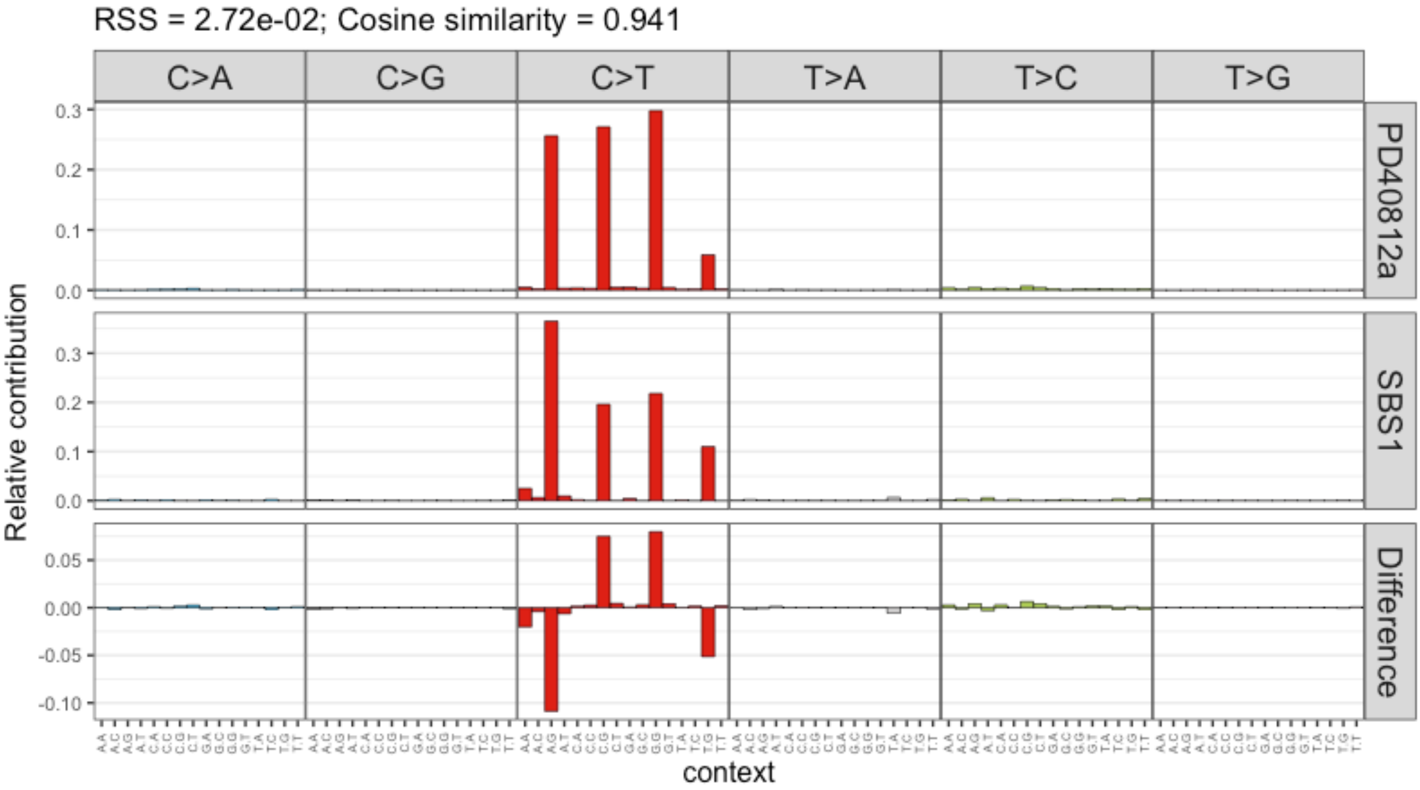

b

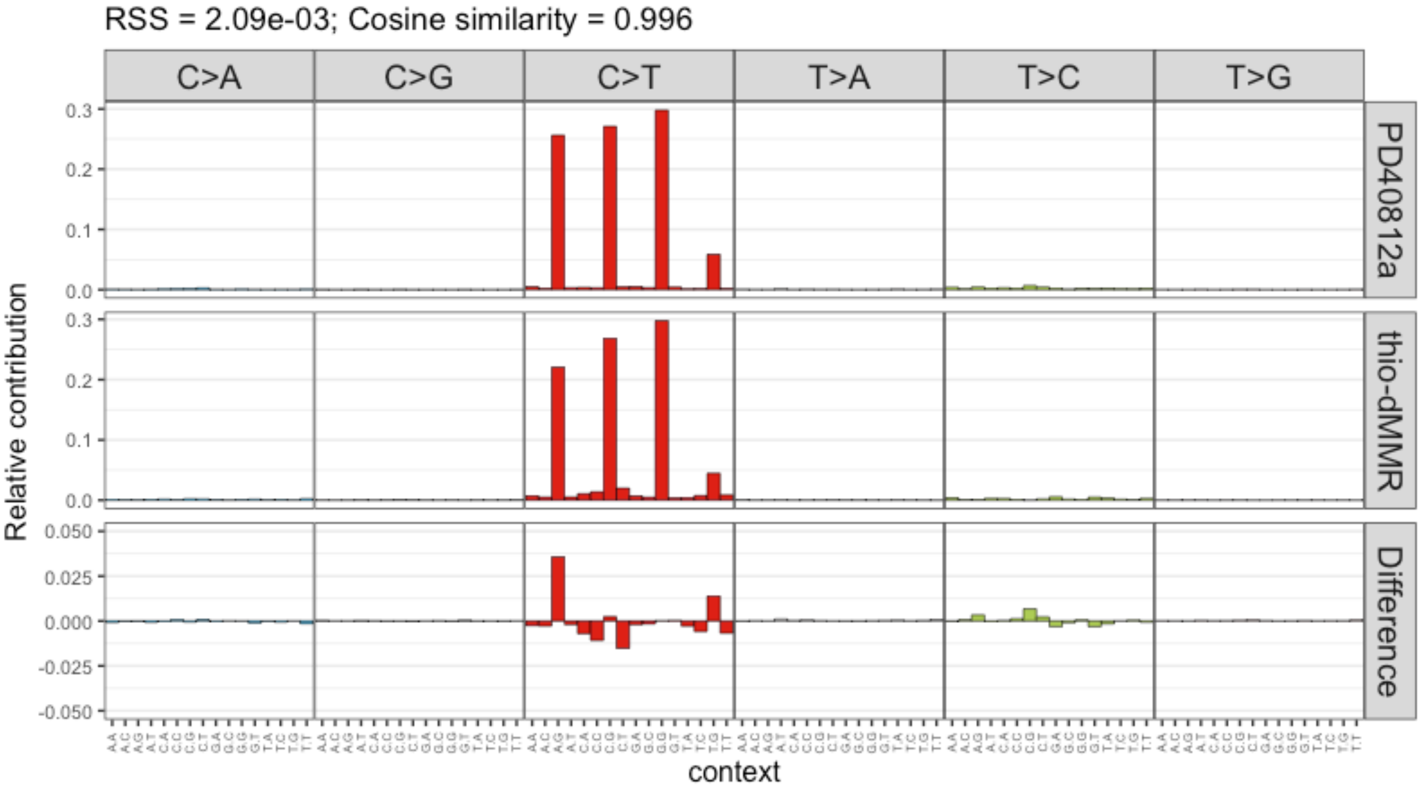

Legend: 96 context mutational profiles of *IGH::DUX4* hypermutator (PD40812) fitted to a) SBS1 (profile from Machado et al. 2022) and b) thio-dMMR (profile from Yang et al. 2021)

Supplemental References

1. Moorman AV, Barretta E, Butler ER, et al. Prognostic impact of chromosomal abnormalities and copy number alterations in adult B-cell precursor acute lymphoblastic leukaemia: a UKALL14 study. *Leukemia*. 2022;36(3):625–636.
2. Schwab CJ, Jones LR, Morrison H, et al. Evaluation of multiplex ligation-dependent probe amplification as a method for the detection of copy number abnormalities in B-cell precursor acute lymphoblastic leukemia. *Genes Chromosomes Cancer*. 2010;49(12):1104–1113.
3. Coffa J, van den Berg J. Analysis of MLPA data using novel software coffalyser.NET by MRC-Holland. *Modern Approaches To Quality Control*. 2011;
4. Pedersen BS, Bhetariya PJ, Brown J, et al. Somalier: rapid relatedness estimation for cancer and germline studies using efficient genome sketches. *Genome Med.* 2020;12(1):62.
5. Jones D, Raine KM, Davies H, et al. cgpCaVEManWrapper: Simple Execution of CaVEMan in Order to Detect Somatic Single Nucleotide Variants in NGS Data. *Curr. Protoc. Bioinformatics*. 2016;56:15.10.1–15.10.18.
6. Ye K, Schulz MH, Long Q, Apweiler R, Ning Z. Pindel: a pattern growth approach to detect break points of large deletions and medium sized insertions from paired-end short reads. *Bioinformatics*. 2009;25(21):2865–2871.
7. Raine KM, Van Loo P, Wedge DC, et al. ascatNgs: Identifying Somatically Acquired Copy-Number Alterations from Whole-Genome Sequencing Data. *Curr. Protoc. Bioinformatics*. 2016;56:15.9.1–15.9.17.
8. Nik-Zainal S, Van Loo P, Wedge DC, et al. The life history of 21 breast cancers. *Cell*. 2012;149(5):994–1007.
9. Nik-Zainal S, Davies H, Staaf J, et al. Landscape of somatic mutations in 560 breast cancer whole-genome sequences. *Nature*. 2016;534(7605):47–54.
10. Mitchell J, Bartram J, Walker S, et al. Clinical application of tumour in normal contamination assessment from whole genome sequencing. *bioRxiv*. 2022;2022.03.09.483623.
11. Chakravarty D, Gao J, Phillips SM, et al. OncoKB: A Precision Oncology Knowledge Base. *JCO Precis Oncol*. 2017;2017.:
12. Robinson JT, Thorvaldsdóttir H, Winckler W, et al. Integrative genomics viewer. *Nat. Biotechnol.* 2011;29(1):24–26.
13. Lilljebjörn H, Fioretos T. New oncogenic subtypes in pediatric B-cell precursor acute lymphoblastic leukemia. *Blood*. 2017;130(12):1395–1401.
14. Roberts KG, Gu Z, Payne-Turner D, et al. High Frequency and Poor Outcome of Philadelphia Chromosome-Like Acute Lymphoblastic Leukemia in Adults. *J. Clin. Oncol.* 2017;35(4):394–401.
15. Kimura S, Montefiori L, Iacobucci I, et al. Enhancer retargeting of CDX2 and UBTF::ATXN7L3 define a subtype of high-risk B-progenitor acute lymphoblastic leukemia. *Blood*. 2022;139(24):3519–3531.
16. Hnisz D, Abraham BJ, Lee TI, et al. Super-enhancers in the control of cell identity and disease. *Cell*. 2013;155(4):934–947.
17. Frankish A, Diekhans M, Ferreira A-M, et al. GENCODE reference annotation for the human and mouse genomes. *Nucleic Acids Res.* 2019;47(D1):D766–D773.
18. Huret J-L, Ahmad M, Arsaban M, et al. Atlas of genetics and cytogenetics in oncology and haematology in 2013. *Nucleic Acids Res.* 2013;41(Database issue):D920–4.
19. Mitelman F JBAMF (eds). Mitelman Database of Chromosome Aberrations and Gene Fusions in Cancer (2022).
20. Shukla N, Levine MF, Gundem G, et al. Feasibility of whole genome and transcriptome profiling in pediatric and young adult cancers. *Nat. Commun.* 2022;13(1):2485.

21. Landrum MJ, Lee JM, Benson M, et al. ClinVar: improving access to variant interpretations and supporting evidence. *Nucleic Acids Res.* 2018;46(D1):D1062–D1067.
22. Karczewski KJ, Francioli LC, Tiao G, et al. Author Correction: The mutational constraint spectrum quantified from variation in 141,456 humans. *Nature*. 2021;590(7846):E53.
23. Blokzijl F, Janssen R, van Boxtel R, Cuppen E. MutationalPatterns: comprehensive genome-wide analysis of mutational processes. *Genome Med.* 2018;10(1):33.
24. Manders F, Brandsma AM, de Kanter J, et al. MutationalPatterns: the one stop shop for the analysis of mutational processes. *BMC Genomics*. 2022;23(1):134.
25. Machado HE, Mitchell E, Øbro NF, et al. Diverse mutational landscapes in human lymphocytes. *Nature*. 2022;
26. Yang F, Brady SW, Tang C, et al. Chemotherapy and mismatch repair deficiency cooperate to fuel TP53 mutagenesis and ALL relapse. *Nature Cancer*. 2021;2(8):819–834.
27. Papaemmanuil E, Rapado I, Li Y, et al. RAG-mediated recombination is the predominant driver of oncogenic rearrangement in ETV6-RUNX1 acute lymphoblastic leukemia. *Nat. Genet.* 2014;46(2):116–125.
28. Farmery JHR, Diseases NB-R, Smith ML, Lynch AG. Telomerecat: A ploidy-agnostic method for estimating telomere length from whole genome sequencing data. *Scientific Reports*. 2018;8(1.):
29. Dobin A, Davis CA, Schlesinger F, et al. STAR: ultrafast universal RNA-seq aligner. *Bioinformatics*. 2013;29(1):15–21.
30. Schmidt B, Brown LM, Ryland GL, et al. ALLSorts: a RNA-Seq classifier for B-Cell Acute Lymphoblastic Leukemia. *bioRxiv*. 2021;2021.08.01.454393.
31. Mäkinen V-P, Rehn J, Breen J, Yeung D, White DL. Multi-Cohort Transcriptomic Subtyping of B-Cell Acute Lymphoblastic Leukemia. *Int. J. Mol. Sci.* 2022;23(9.):
32. Morales J, Pujar S, Loveland JE, et al. A joint NCBI and EMBL-EBI transcript set for clinical genomics and research. *Nature*. 2022;604(7905):310–315.
